# Enhancing Interlayer Bonding in DLP‐Printed Piezoelectric Ceramics via Controlled Secondary Curing for High Piezoelectric Performance

**DOI:** 10.1002/advs.202512767

**Published:** 2025-09-04

**Authors:** Yaoting Zhao, Ruihang Liu, Wenlong Wang, Jin Zhang, Hongya Liu, Wei Gao, Xiujuan Lin, Hang Luo, Shifeng Huang, Dou Zhang

**Affiliations:** ^1^ Shandong Provincial Key Laboratory of Green and Intelligent Building Materials University of Jinan Jinan 250022 China; ^2^ Naval Submarine Academy Qingdao 266199 China; ^3^ State Key Laboratory of Powder Metallurgy Central South University Changsha 410083 China

**Keywords:** digital light processing, interlayer bonding, piezoelectric ceramic, secondary curing

## Abstract

Digital light processing (DLP) presents a promising approach for fabricating intricately designed piezoelectric components, which are essential for developing high‐sensitivity piezoelectric sensor systems. However, the inherent layer‐by‐layer stacking nature of DLP induces interlayer cracking in printed ceramics, which severely deteriorates their performance. This work introduces an innovative interfacial engineering strategy to print superlattice components with exceptional piezoelectric performance. A moderate exposure time improves the surface characteristics of cured layers, that initiates controlled secondary curing at material interfaces. This process strengthens interlayer bonding and consequently boosts the mechanical properties of the green body. The optimal interlayer bonding quality is observed for t‐20 ceramics. Its piezoelectric constant (*d_33_*) reaches an exciting 516 ± 8 pC·N^−1^, which approaches the level of commercial ceramic and exceeds the values of most 3D‐printed advanced piezoelectric materials. The printed superlattice component achieves an ultra‐high piezoelectric response, e.g., open‐circuit voltage up to 493 V at 17.3 N. The component exhibits an extremely pressure‐sensitive sensitivity of 27.9 V·N^−1^, along with a voltage response at a very faint load of 0.1 N. Applications in various scenarios show the component's favorable sensing characteristics and reliable non‐contact dynamic monitoring capability, which opens new avenues for its applications in the sensing field.

## Graphical abstract

This study introduces an interfacial secondary curing strategy to overcome interlayer delamination in DLP‐printed PZT ceramics. Precise exposure time modulation enhances interfacial bonding via unsaturated bond conversion and stress control. Printed components exhibit a high piezoelectric coefficient (*d_33_* = 516 pC·N^−1^) and exceptional sensing performance (27.9 V·N^−1^). This approach enables robust piezoelectric sensors for high‐sensitivity detection and non‐contact monitoring applications.

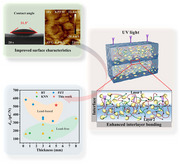

## 1 Introduction

Lead zirconate titanate (PbTiZrO_3_, PZT) piezoelectric ceramics, as a class of high‐performance smart materials, have found extensive applications in sensing,^[^ ^1^ ^]^ energy harvesting,^[^ ^2^ ^]^ and actuators^[^ ^3^ ^]^ owing to their exceptional electromechanical response characteristics and integrated sensing‐actuation functionality. Nevertheless, the inherent brittleness of ceramic materials presents significant challenges in the fabrication of high‐precision components with complex geometries.^[^ ^4^, ^5^ ^]^ Moreover, mechanical stresses induced during processing are known to compromise the structural integrity of ceramics and induce near‐surface depolarization, which critically impedes the advancement of piezoelectric ceramic technologies.^[^ ^6^ ^]^

Additive manufacturing (AM) represents a cutting‐edge material‐forming technology that has garnered substantial attention across both academia and industry since its emergence.^[^ ^7^, ^8^ ^]^ Among various AM techniques, digital light processing (DLP), a photopolymerization‐based method, has gained prominence in the fabrication of piezoelectric ceramics due to its great printing resolution, superior surface quality, and high processing efficiency.^[^ ^9^, ^10^, ^11^ ^]^ This approach enables the tailored production of intricate ceramic structures with high dimensional precision.^[^ ^12^, ^13^, ^14^ ^]^ In DLP technology, ceramic slurry is selectively cured via digital UV irradiation, followed by layer‐by‐layer stacking to achieve components with complex geometries. However, the inherent layer‐wise stacking mechanism introduces interlayer cracks as a predominant defect during printing. The resulting weak interlayer bonding significantly degrades the mechanical and functional performance of the printed ceramics, posing a critical challenge for practical applications.

The key printing parameters in light‐curing 3D printing technology primarily include curing depth, layer thickness, exposure power, and exposure time.^[^ ^15^ ^]^ Considerable research efforts have been devoted to optimizing layer thickness modulation during printing to improve interlayer bonding quality and reduce interlayer defects.^[^ ^16^, ^17^, ^18^, ^19^, ^20^ ^]^ For instance, Mohammadi et al.^[^ ^19^ ^]^ attained favorable interlayer bonding quality in printed samples when the curing depth to cured layer thickness ratio of high‐strength hydroxyapatite ceramics was regulated at 1.6. Li et al.^[^ ^20^ ^]^ investigated the influence of curing depth on the formability quality of printed alumina ceramics, and found that increasing the curing depth improved the interlayer bonding. Huang et al.^[^ ^21^ ^]^ addressed the prevalent delamination issue in silicon nitride honeycomb ceramics by augmenting the photoinitiator concentration to increase slurry curing depth. Nevertheless, excessive curing depth can induce substantial internal stresses within green bodies, consequently promoting interlayer defect formation.^[^ ^22^, ^23^ ^]^ Chartier et al.^[^ ^24^ ^]^ established a critical relationship between monomer conversion rate and cured layer stresses, proposing that a multi‐stage curing strategy with reduced energy density could promote more homogeneous polymerization and thereby strengthen interlayer bonding. Furthermore, an overly aggressive curing depth has been shown to adversely affect debinding and pyrolysis processes, potentially introducing defects in the final sintered ceramics.^[^ ^25^, ^26^ ^]^

An increase in curing depth is closely associated with an augmented in exposure energy, which is determined by exposure power and exposure time. Given the scattering effect of ceramic particles on UV light, merely increasing exposure power to achieve greater curing depth substantially compromises printing accuracy.^[^ ^27^, ^28^ ^]^ This issue is particularly pronounced for PZT and barium titanate (BaTiO_3_, BT) piezoelectric ceramics, which demonstrate heightened sensitivity to UV scattering effects. Consequently, these materials are prone to severe overcuring at elevated exposure powers, potentially inducing interlayer defects. To mitigate these issues, Liu et al.^[^ ^29^ ^]^ explored the incorporation of 50 vol% soluble starch into PZT slurry to enhance interlayer bonding. Yet, the presence of soluble starch results in a low relative density of 89.51% and diminished piezoelectric properties, with a *d_33_* of only 373 pC·N^−1^. Alternatively, maintaining constant energy input while compensating for reduced power through extended exposure time presents a viable strategy for effective slurry printing. Liu et al.^[^ ^30^ ^]^ improved the interlayer bonding of BT ceramics by designing the structures of adhesion, transition, and main printing layers and optimizing the printing parameters of each layer (e.g., exposure time). This methodology achieved great material properties, with relative density reaching 95.32% and piezoelectric constant improving to 168.1 pC·N^−1^, underscoring the critical role of exposure time in interfacial quality. Notably, the fundamental mechanisms governing interlayer strengthening through exposure time control remain insufficiently explored. Particularly, interfacial enhancement mechanisms such as unsaturated double bond conversion in resin monomers and interfacial secondary curing warrant in‐depth investigation to establish comprehensive processing‐structure‐property relationships.

This study proposes an innovative strategy to significantly improve interlayer bonding quality in DLP‐printed PZT piezoelectric ceramics through triggered controllable interface secondary curing, ultimately yielding piezoelectric components with excellent electromechanical performance. The relationship between slurry curing behavior and cured layer surface characteristics was systematically investigated by precisely modulating exposure time parameters. To fundamentally understand the interlayer strengthening mechanism, a comprehensive analysis was conducted on the double bond conversion kinetics and their direct impact on interfacial bonding quality. These mechanistic studies were rigorously validated through combined microstructural characterization, electrical property measurements, and. The resulting piezoelectric components demonstrated exceptional sensing performance and reliable non‐contact dynamic monitoring capabilities, as evidenced by performance evaluations. These findings provide critical insights and establish practical guidelines for enhancing interfacial integrity in 3D‐printed functional ceramics, offering a viable pathway for performance optimization in advanced piezoelectric applications.

## 2 Results and Discussion

### 2.1 Slurry Performance and Cured Layer Surface Characteristics

**Figure 1:**
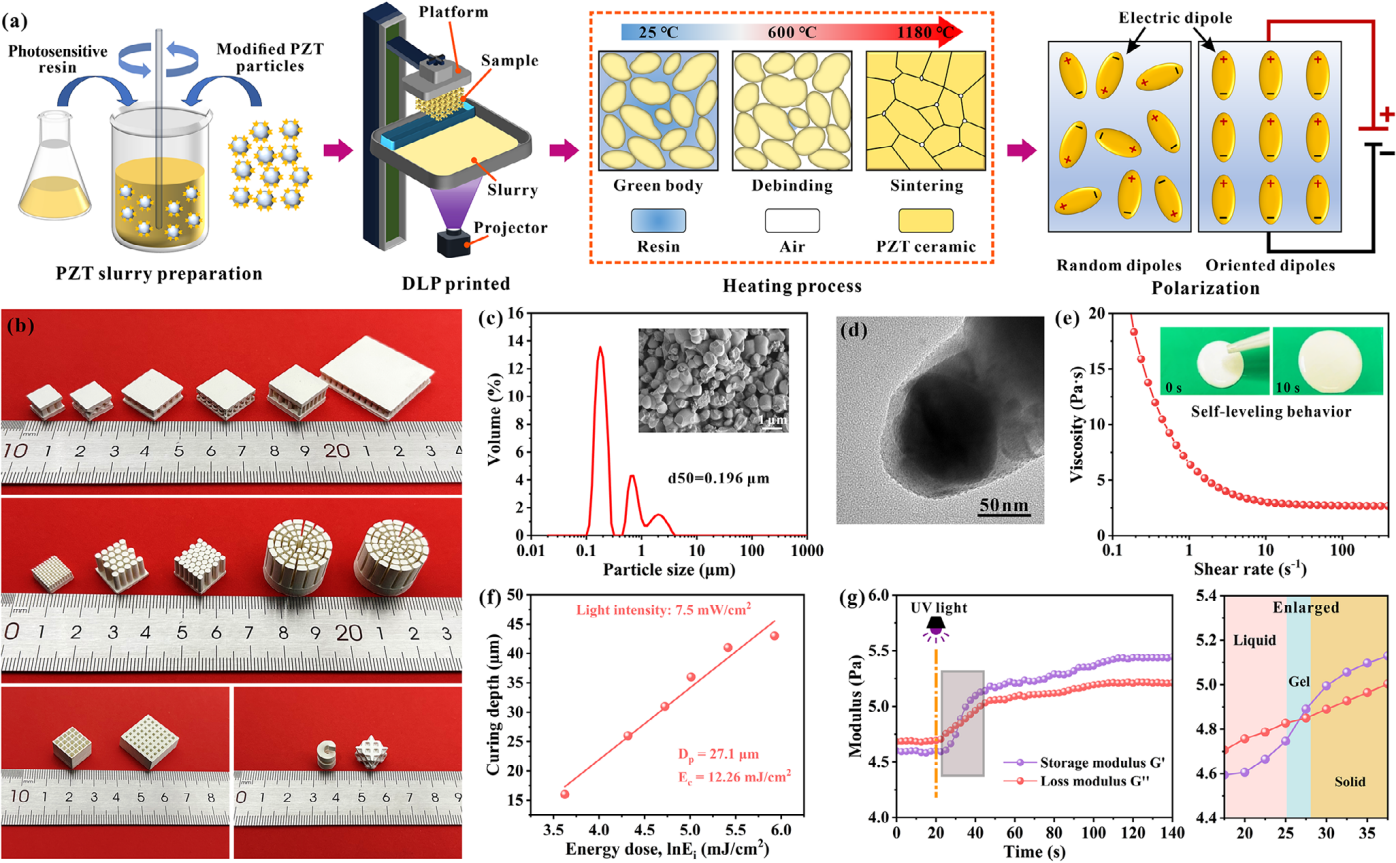
Preparation of PZT ceramic components. a) Manufacturing process for DLP‐printed ceramic components. b) Printed components with different structures. c) Morphology and particle size distribution of PZT powder. d) TEM image of modified powder. e) Viscosity and self‐leveling behaviors of PZT slurry. f) Curing properties of the slurry at different exposure energies. g) Rheological characterization of slurries under UV irradiation.

As illustrated in **Figure** **1**a, the optimized PZT ceramic slurry was precisely fabricated into pre‐designed 3D architectures using DLP‐based additive manufacturing. The as‐printed green bodies subsequently underwent thermal debinding and sintering processes to yield densified and structurally integrated ceramic components. Following sintering, the ceramic components were electrically polarized to activate their piezoelectric functionality. Figure 1b highlights the remarkable geometric versatility afforded by DLP technology, showcasing an array of intricately designed architectures tailored for diverse functional applications. These include a meticulously engineered superlattice structure optimized for high‐sensitivity piezoelectric sensing, precisely arranged 1‐3‐2 and 3‐1 periodic arrays designed to enhance transducer efficiency, and an advanced helical configuration strategically developed to maximize actuation performance.

To minimize sintering‐induced shrinkage and maintain exceptional dimensional fidelity, a slurry with ultra‐high solid loading (60 vol% or 91 wt%) is formulated for printing. This was achieved through a multifaceted optimization strategy, which involved precise particle grading of PZT powders to maximize solid loading (Figure 1c) while simultaneously leveraging BYK‐111 dispersant‐modified particles to significantly reduce slurry viscosity (Figure 1d). The XRD results (Figure S1, Supporting Information) confirm that the modified PZT powder still retains a well‐defined perovskite structure with high crystallinity. As evidenced in Figure 1e and Figure S2 (Supporting Information), the optimized slurry exhibited outstanding self‐leveling behavior, with a measured viscosity of 2.71 Pa·s at a shear rate of 100 s^−1^, well below the critical 3 Pa·s threshold required for high fidelity DLP printing.^[^ ^31^ ^]^ The curing behavior of the slurry, a vital factor governing printing accuracy and interlayer bonding, was quantitatively analyzed via the Beer‐Lambert model (Equation S1, Supporting Information). Figure 1f illustrates a relationship between penetration depth (*D_p_*) and the logarithmic incident energy (*E_c_*), with an optimal curing depth of 27.1 µm achieved at an energy dosage of 12.26 mJ·cm^−2^. Beyond slurry formulation, light‐curing parameters, particularly exposure time, play a decisive role in determining the structural integrity and functional performance of the printed materials and components. In Figure 1g, the photopolymerization kinetics of the slurry were investigated by in situ photorheological measurements. Following an initial 20 s stabilization period, UV irradiation triggered rapid crosslinking of resin, with the gel point attained within just 7 s as evidenced by the crossover of storage modulus (*G'*) and loss modulus (*G″*). Although prolonged exposure further enhanced mechanical properties, the modulus values stabilized after 40 s, confirming that the slurry had reached its maximum degree of polymerization.

**Figure 2:**
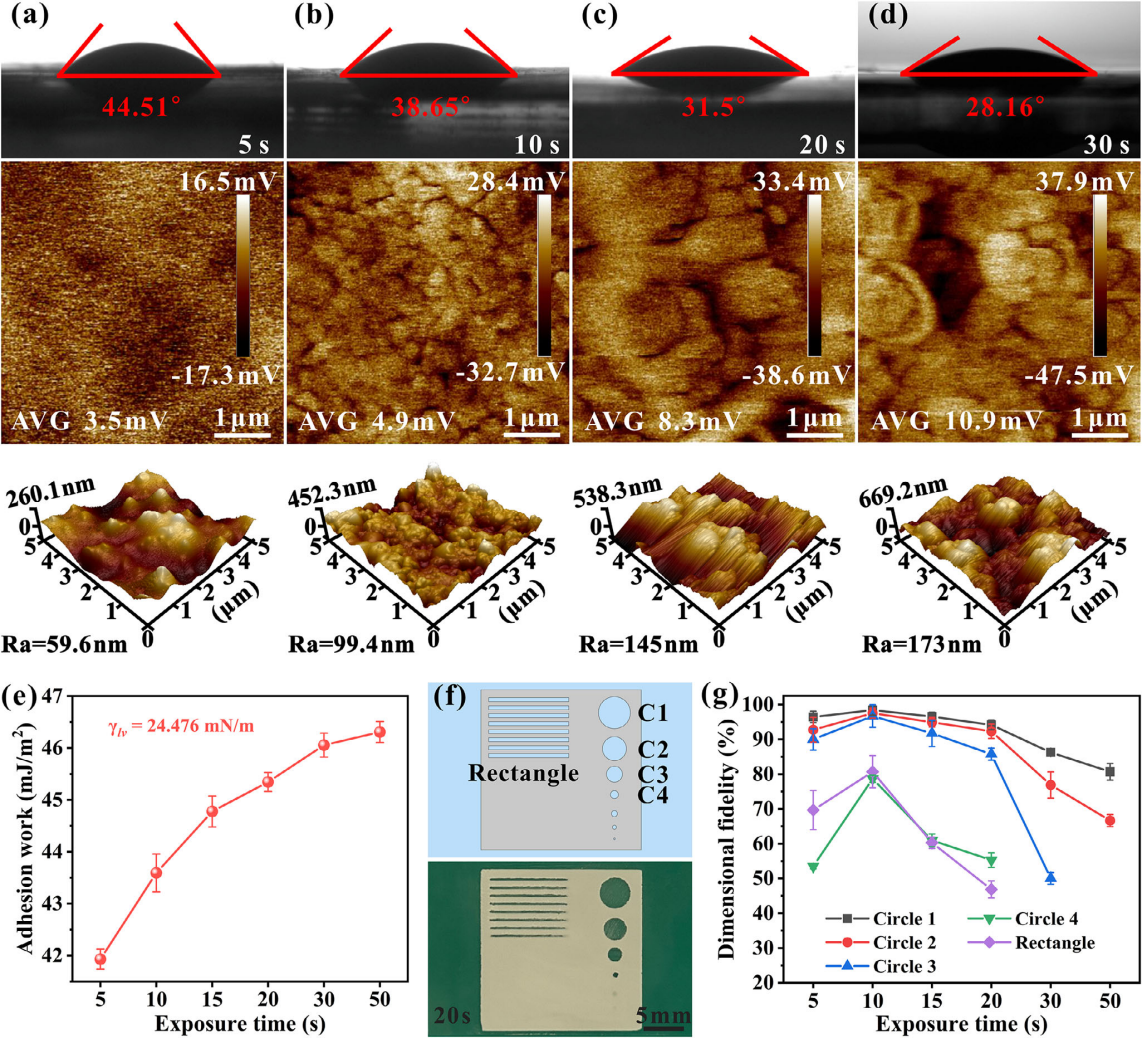
Surface characterization of the cured layer for PZT slurry. a–d) Contact angle, surface potential, and surface roughness of cured layers at different exposure times. e) Adhesion work (*W_a_*) on the cured layer surface. f,g) Printing accuracy and dimensional fidelity of the cured layers.

DLP printing of ceramics is a layer‐by‐layer stacking additive manufacturing process wherein each newly cured layer undergoes photopolymerization while submerged in the uncured slurry. It is worth noting that the surface characteristics of previously cured layers exhibit dynamic evolution with progressive UV exposure, as evidenced by corresponding changes in the elastic modulus of the polymerized slurry. This time‐dependent modification induces a wettability gradient at the slurry‐cured layer interface, with significant implications for interlayer bonding quality. The contact angle (CA) measurements in **Figures** **2**a–d and S3 (Supporting Information) demonstrate the enhanced wettability of the slurry on the cured layer surface with an increase in exposure time, and the CA decreases from 44.51° to 28.16°. This enhanced wettability contributes to the spreading of the slurry on the cured layer surface during the printing process, effectively mitigating interlayer defect formation. The observed CA variation correlates strongly with surface potential alterations, as confirmed by Kelvin probe force microscopy (KPFM) measurements demonstrating a surface potential increase from 3.5 to 10.9 mV. This elevation in surface potential directly corresponds to increased surface energy, which is well‐established to inversely correlate with contact angle and positively influence wettability.^[^ ^32^ ^]^ Adhesion work (*W_a_*), a critical metric for interlayer bonding strength,^[^ ^33^, ^34^ ^]^ was quantitatively determined through contact angle analysis using Equation (1):

(1) $$W_{a}=\gamma_{lv}\;\left(1+cos\theta \right)$$

here, *γ_lv_* stands for the surface tension of slurry, and *θ* denotes the measured contact angle. As illustrated in Figure 2e, prolonged exposure time leads to a marked increase in adhesion work from 41.93 to 46.05 mJ·m^−2^, indicating strengthened interfacial bonding between successive layers. This enhancement in adhesion work significantly improves interlayer bonding, thereby optimizing the structural integrity of the printed component. In addition, surface topography analysis of the cured layers reveals that extending the exposure time from 5 to 50 s results in a 113.6 nm increase in surface roughness (*R_a_*). While elevated roughness may theoretically compromise printing resolution, these research findings demonstrate that optimal exposure parameters must carefully balance competing factors: sufficient curing depth for robust interlayer bonding versus minimal overcuring for dimensional accuracy. In Figure 2f, an accuracy testing model was designed that includes rectangles and circles of different sizes. Only four circles of different diameters can appear on the cured layer, which are named C1 (d = 4 mm), C2 (d = 2 mm), C3 (d = 1 mm), and C4 (d = 0.5 mm). Figure S4 (Supporting Information) demonstrates that shorter exposure time (5 s) yields superior printing accuracy; however, insufficient curing depth at this stage compromises interlayer bonding and induces wrinkling defects due to shrinkage stress. Conversely, excessive exposure time (50 s) leads to pronounced overcuring, severely degrading printing precision beyond acceptable DLP printing standards. The dimensional fidelity of the cured layer shown in Figure 2g confirms this. Prolonged exposure times significantly compromise dimensional fidelity, particularly when the time exceeds 20 s, with a sharp decline in fidelity observed for circles C1‐C3, and circle C4, and the rectangle being completely covered by the over‐cured layer. Notably, the 10 s cured layer exhibits marginally superior dimensional accuracy compared to their 5 s counterparts, where surface wrinkling‐induced distortions exacerbate measurement errors. Despite minor overcuring effects at 20 s exposure time, the structural fidelity remains sufficient to clearly resolve the three distinct circles. Given the macroscopic scale of the printed ceramics, these subtle deviations are negligible and do not adversely affect overall performance.

### 2.2 Interfacial Mechanical Properties of Green Body

**Figure 3:**
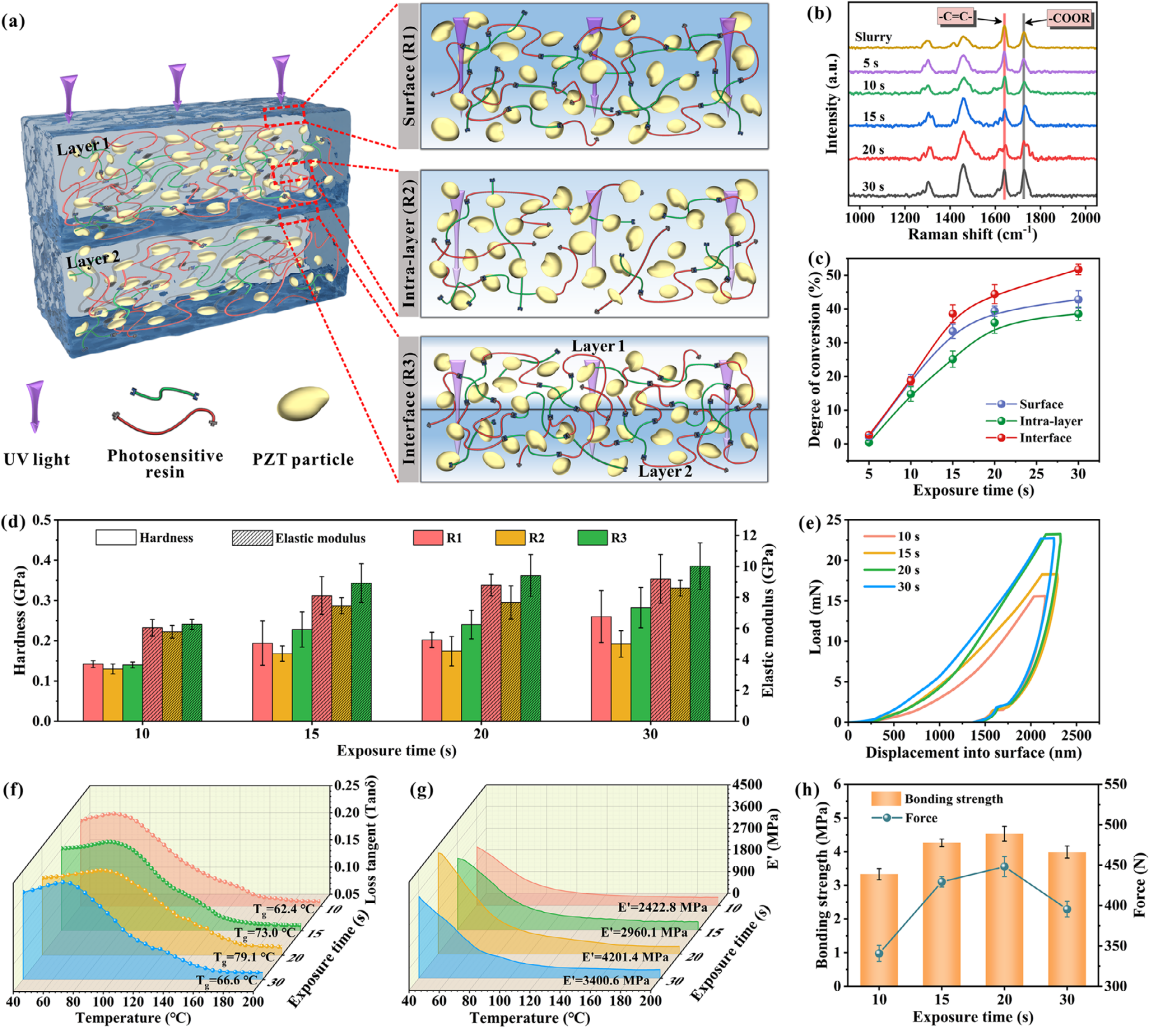
Mechanical properties of printed PZT green bodies. a) Schematic diagram of the cross‐sectional structure of the green body. b) Raman spectra at R3. c) Unsaturated double bond conversion ratio as well as d) hardness and elastic modulus at different regions. e) Displacement‐load curve for R3. f) Mechanical loss tangent (*Tanδ*), g) storage modulus E', and h) interlayer bonding strength of the green bodies.

The UV light scattering effect induced by ceramic particles within the slurry leads to progressive diminution of radiation intensity with increasing penetration depth. This optical attenuation phenomenon directly influences the polymerization kinetics, creating substantial depth‐dependent variations in the conversion rate of unsaturated double bonds within the photosensitive resin matrix. In consideration of the discrepancy in conversion rates, the cured layer was segmented into three distinct regions: the surface (R1), the intra‐layer (R2), and the interface between two layers (R3), as seen in **Figure** **3**a. Raman spectroscopic analysis of the green body cross‐sections in Figure 3b and Figure S5 (Supporting Information) identified characteristic vibrational modes at 1636 cm^−1^ (corresponding to C═C stretching) and 1720 cm^−1^ (associated with C═O stretching of ester groups). The unsaturated double bond conversion rate was calculated by the Lorentzian function (Equation (2)):^[^ ^24^ ^]^

(2) $$C_{Raman}\;\left(\%\right)=\frac{\frac{A_{0}^{1636}}{A_{0}^{1720}}\;-\;\frac{A_{end}^{1636}}{A_{end}^{1720}}}{\frac{A_{0}^{1636}}{A_{0}^{1720}}}\;\times 100$$

where *A_0_* is the peak area measured on the suspension before any UV exposure, and *A_end_* is the peak area measured on the green body. The area *A_1636_* corresponds to the area measured in the 1600–1650 cm^−1^ region, while the area *A_1720_* corresponds to the area measured in the 1650–1780 cm^−1^ region. The conversion rate depicted in Figure 3c reveals a favorable spatial dependence, with R3 exhibiting the highest conversion (over 44% at 20 s), followed by R1, while R2 consistently showed the lowest values. The FTIR results in Figure S6 (Supporting Information) also confirm this. This interfacial enhancement at R3 originates from synergistic secondary curing between residual unconverted double bonds in previously cured layers and freshly polymerized material, enabled by sufficient curing depth of the slurry. This dual‐curing mechanism substantially enhances resin crosslink density at interfaces, a process that is effective in promoting interlayer bonding. Notably, this gradient effect exhibits pronounced exposure time dependence, becoming increasingly significant with longer curing durations. However, it is noteworthy that there is no significant difference between the different regions of the t‐10 green body. This is attributable to the inferior curing depth of 26 µm, which is insufficient to induce secondary curing of the preceding cured layer. Such inadequate curing can readily result in defects, including interlayer cracks. The presence of interlayer defects has been demonstrated to be a primary factor contributing to the deterioration of the mechanical properties of the printed green bodies. In Figure 3d, the nanoindentation results indicate that the largest values of hardness and elastic modulus were observed at R3 within the same green body. Furthermore, the extended exposure time led to an increase in the overall hardness and modulus of the green body, which has reached 0.24 ± 0.04 GPa and 9.4 ± 1.3 GPa in just 20 s. The resistance to plastic deformation of the green bodies was further analyzed by the discussion of displacement‐load curves, as demonstrated in Figure 3e and Figure S7 (Supporting Information). It is determined that the load necessary for the indenter to press into a depth of 2 µm at R3 in the t‐20 and t‐30 green bodies is nearly equivalent. This phenomenon can be attributed to the increase in crosslink density resulting from the elevated level of double bond conversion within the resin. Dynamic thermo‐mechanical (DMA) analysis in Figure 3f,g corroborates these findings, with the t‐20 green body displaying optimal thermomechanical performance, minimal mechanical loss, highest glass transition temperature of 73.01 °C, and maximum storage modulus *E'* of 4201.4 MPa. These characteristics confirm robust interfacial bonding between each layer in the green body, thereby resulting in enhanced its thermo‐mechanical properties. The interlayer bonding strength of the printed green body was quantitatively evaluated via tensile testing (Figure 3h). The bonding strength demonstrates a non‐monotonic dependence on exposure time, peaking at 4.53 MPa for the t‐20 green body. Corresponding tensile testing curves in Figure S8 (Supporting Information) reveal that the t‐20 green body achieves a maximum load‐bearing capacity of 448.07 N, further confirming the superior interfacial integrity of this green body. While the t‐30 green body showed a decrease in properties, perhaps related to its interlayer defects. Excessive conversion rates can result in severe resin curing shrinkage and elevated internal stresses within the green bodies.^[^ ^35^, ^36^ ^]^ As demonstrated in **Table** **1**, peak positions in Raman spectra shift toward higher wavenumbers with increased exposure time, further evidence these internal stresses. An increase in shrinkage stresses within the cured layer leads to cracking at the interlayer interface, thus deteriorate the mechanical properties of green bodies.

**Table 1:** Raman peak shifts on the cured layer surface.

Times [s]	Peak position [cm^−1^]			
0	1299	1456	1636	1720
5	1.25	0.98	0	2.03
10	1.83	1.57	1.68	3.12
15	2.39	2.26	2.53	3.52
20	4.79	4.57	3.53	4.23
30	4.72	4.46	3.76	4.43

Finite element analysis (FEA) was employed to investigate the interfacial bonding characteristics of printed green bodies in Figure S9 (Supporting Information). The simulation yields quantitative distributions of interfacial shear strength and residual stress. As illustrated in Figure S9a (Supporting Information), interfacial shear strength initially increases with exposure time, peaking at 20 s where optimal interlayer bonding is achieved. Beyond this threshold, the diminishing rate of elastic modulus increase (Figure 3d) correlates with reduced shear stress and consequent weakening of interfacial strength. Furthermore, residual stress accumulation in the cured layer adversely affects interlayer bonding. UV‐induced polymerization shrinkage generates substantial residual stresses, whose magnitude escalates progressively with exposure time (Figure S9b, Supporting Information). These stresses induce shrinkage deformation, promoting defect formation such as interlayer delamination and consequently compromising bonding integrity. These computational results exhibit excellent consistency with the experimental observations presented in Figure 3f–h.

### 2.3 Microstructure and Electrical Performance of Ceramics

**Figure 4:**
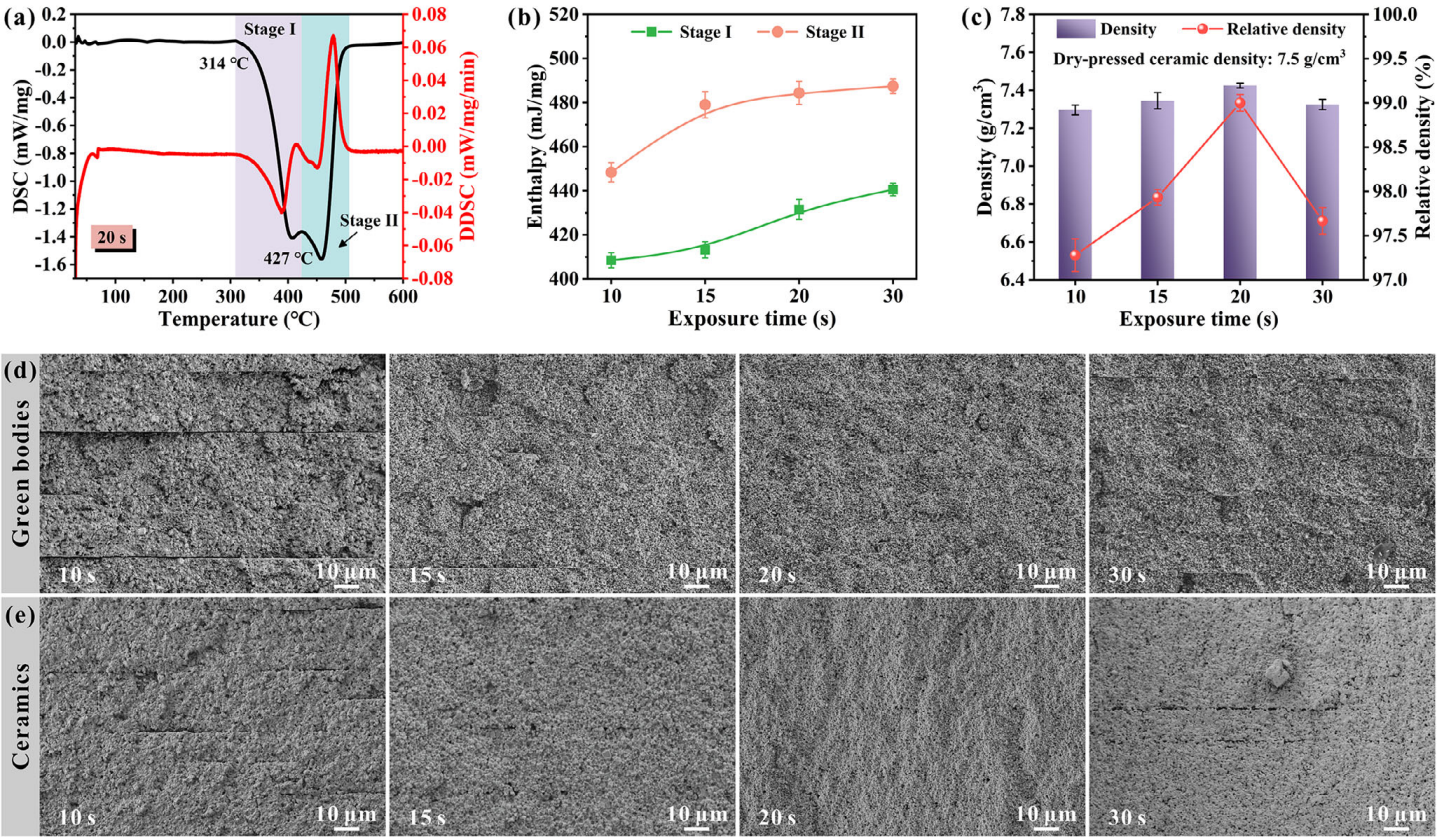
Exothermic behavior analysis and microstructures of printed ceramics. a) DSC‐DDSC curves of t‐20 green body. b) Heat release of the two stages. c) Density of printed ceramics. d) Cross‐sectional SEM images of green bodies and e) the corresponding sintered ceramics.

The modulation of exposure time exerts a significant influence on the photopolymerization kinetics of the photosensitive resin system, which subsequently affects its pyrolytic decomposition characteristics during the thermal debinding process. Comprehensive thermal analysis conducted via thermogravimetric (TG) in Figure S10a–d (Supporting Information) reveals two well‐defined pyrolysis stages, designated as stage I (low‐temperature decomposition) and stage II (high‐temperature decomposition). As illustrated in Figure S10e (Supporting Information), the percentage of mass loss in each stage indicates that the majority of the mass loss occurred in stage I, which accounted for more than 80% of the total mass loss. In contrast, stage II accounted for less than 20% of the total mass loss. This observation suggests that the majority of the resins underwent pyrolysis during stage I, with only a minor proportion being fully pyrolyzed during stage II. This can also be confirmed by the differential scanning calorimetry (DSC) results in **Figures** **4**a, and S11a–c (Supporting Information). As demonstrated in Figures S10f and S11d (Supporting Information), the onset temperature of stage II pyrolysis exhibits a positive correlation with exposure time, progressively increasing from 452 to 463 °C (from 422 °C to 431 °C in DSC) as exposure time is extended. This thermally stabilizing effect can be mechanistically attributed to the exposure time‐dependent evolution of resin composition. Prolonged UV irradiation facilitates more complete consumption of unsaturated double bonds through crosslinking reactions, thereby yielding a polymer network with enhanced thermal stability that requires higher energy input for thermal decomposition.^[^ ^20^ ^]^ It is noteworthy that the integrated heat flow analysis presented in Figure 4b demonstrates that both pyrolysis stages exhibit time‐dependent increases in enthalpy release, with stage I consistently displaying slightly lower heat evolution compared to stage II. This controlled energy dissipation profile is particularly advantageous as it prevents abrupt heat release that could lead to localized thermal accumulation, facilitates gradual removal of organic components, and most importantly, effectively mitigates the development of thermal stress‐induced defects such as interlayer delamination or microcrack formation during debinding.

The sintering temperature critically influences the structural and piezoelectric properties of printed ceramics. At the lower temperature of 1160 °C (Figure S12a, Supporting Information), insufficient thermal energy results in limited grain growth, yielding ceramics with finer grains and higher porosity. Conversely, excessively high sintering temperature at 1200 °C (Figure S12c, Supporting Information) causes over‐firing and subsequent performance degradation. The ceramic structure sintered at 1180 °C is relatively dense and exhibits higher piezoelectric properties (Figure S12b,d, Supporting Information). Based on the above analysis, the degreasing and sintering processes are obtained as shown in Figure S12e (Supporting Information).

Microstructural characterization reveals significant variations in ceramic density and dimensional stability as a function of exposure time. As shown in Figure 4c, the t‐20 ceramic specimen achieves exceptional densification with a high density of 7.425 ± 0.013 g·cm^−3^, approaching the theoretical density of PZT ceramics. This optimal performance is further corroborated by dimensional stability measurements in Figure S13 (Supporting Information), which record minimal anisotropic shrinkage of merely 13.2% and 13.4% along the axial and radial directions, respectively. These remarkable characteristics can be attributed to the fundamental relationship between defect minimization and microstructural evolution, the substantial reduction of internal flaws during the printing process facilitates the development of a highly compact ceramic microstructure during sintering. The intrinsic layer‐by‐layer fabrication mode of DLP technology renders interlayer bonding quality the most critical determinant of final ceramic integrity. As seen in Figure 4d, comparative analysis demonstrates that the t‐20 green body has the best interlayer bonding quality, while the t‐10 and t‐30 green bodies exhibit severe interlayer defects. This observation aligns perfectly with previous findings regarding curing mechanisms. The t‐10 green body suffers from insufficient curing depth, resulting in fragile interlayer bonding that propagates as interlayer cracks during thermal processing. And the t‐30 specimen experiences excessive double bond conversion, generating detrimental internal stresses and exaggerated shrinkage that ultimately compromise interfacial integrity. These microstructural observations exhibit well consistency with the mechanical property trends of the green body presented in Figure 3e–h, establishing a favorable structure‐property relationship. The cross‐sectional morphology of sintered ceramics in Figure 4e shows that the t‐20 ceramics have great interlayer bonding quality and a dense structure with a grain size of ≈1.19 µm (Figure S14, Supporting Information). The microstructural advantages of t‐20 ceramics directly manifest in enhanced performance metrics, where the synergistic combination of defect‐free interfaces and homogeneous grain structure accounts for both the achieved near‐theoretical density and remarkably constrained sintering shrinkage. In stark contrast, both t‐10 and t‐30 ceramics display significant interlayer cracking, providing direct evidence that printing‐induced defects become permanently “locked in” during the early fabrication stages and cannot be remedied through subsequent sintering. This irreversible defect incorporation ultimately leads to significant deterioration in the functional performance of the piezoelectric ceramic components.

**Figure 5:**
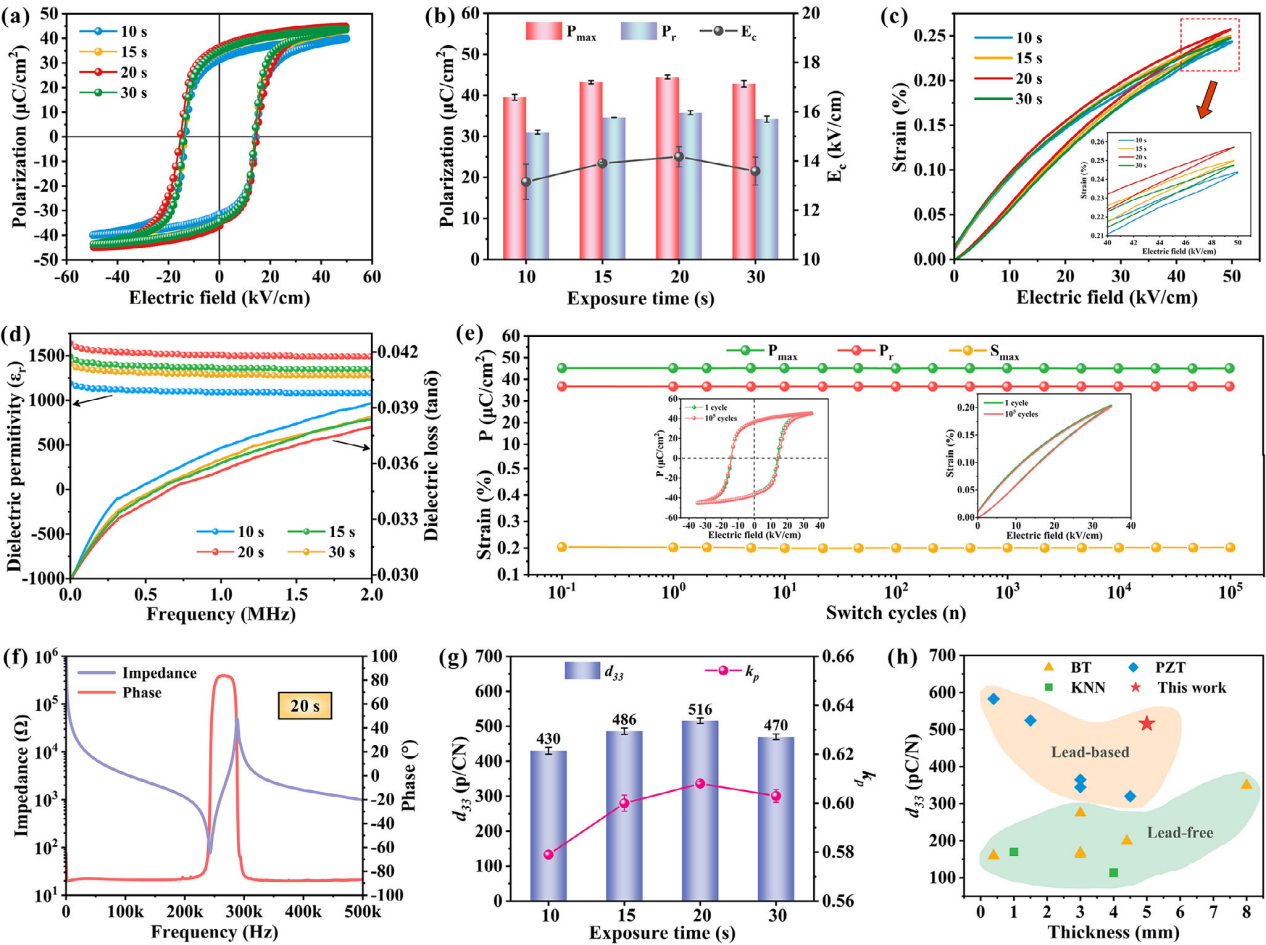
Electrical properties of printed ceramics. a) *P–E* curves. b) *P_max_*, *P_r_*, and *E_c_*. c) *S–E* curves. d) Dielectric constant (*ɛ_r_*) and dielectric loss (t*anδ*). e) Fatigue properties of t‐20 printed ceramic. f) Impedance and phase spectrum. g) Piezoelectric constant (*d_33_*) and electromechanical coupling coefficient (*k_p_*). h) Comparison of 3D‐printed piezoelectric materials.

The electromechanical performance of PZT ceramics is intrinsically governed by their microstructural characteristics, with optimal densification and defect minimization serving as prerequisites for achieving superior properties. Comprehensive investigation of ferroelectric and piezoelectric behavior reveals the t‐20 ceramic exhibits exceptional functional performance. In **Figure** **5**a–c, under a 50 kV·cm^−1^ electric field, the polarization‐electric field (*P‐E*) hysteresis loops and the unipolar strain‐electric field (*S‐E*) curves demonstrate the t‐20 ceramic achieves peak values of maximum polarization (*P_max_* = 44.47 ± 0.47 µC·cm^−2^), remnant polarization (*P_r_* = 35.77 ± 0.47 µC·cm^−2^), and maximum strain (*S_max_* = 0.257%). Especially its strain characteristics approach the theoretical value of pristine ceramic specimens, thereby underscoring its considerable potential for deployment in advanced piezoelectric actuator technologies. And dielectric measurements further confirm the superior performance of the t‐20 ceramics, exhibiting a greatly high relative permittivity (*ε_r_*) of 1720 coupled with remarkably low dielectric loss (*tanδ*) of 0.026 at 1 kHz, as seen in Figure 5d and Figure S15 (Supporting Information). These outstanding dielectric properties ensure minimal energy dissipation, making this material particularly suitable for high‐power device applications where thermal management is critical. The observed property degradation in t‐30 ceramics directly correlates with microstructural defects, particularly interlayer cracking, thereby substantiating the pivotal role of interfacial integrity in determining functional performance. As depicted in Figure 5e, the t‐20 ceramic demonstrates favorable fatigue resistance, maintaining stable *P‐E* and *S‐E* characteristics through 10^5^ cycles at 35 kV·cm^−1^ electric field, with preserved hysteresis loop symmetry, a key indicator of operational reliability in practical applications. In Figure 5f and Figure S16 (Supporting Information), impedance spectroscopy analysis reveals pure electromechanical resonance modes, enabling calculation of an impressive electromechanical coupling coefficient (*k_p_*) of up to 0.608 that signifies highly efficient energy conversion capability. What's even more exciting is that the piezoelectric constant (*d_33_*) of the t‐20 ceramic shown in Figure 5g reaches a remarkable 516 ± 8 pC·N^−1^, which is almost at the level of commercial ceramics (*d_33_* = 530 pC·N^−1^) fabricated by the conventional technologies. In Figure 5h, the ceramic components printed in this study display excellent piezoelectric properties and size advantages in comparison to other 3D‐printed advanced piezoelectric materials.^[^ ^6^, ^29^, ^30^, ^37^, ^38^, ^39^, ^40^, ^41^, ^42^, ^43^, ^44^, ^45^, ^46^ ^]^ While some reported PZT ceramics achieved a higher *d_33_*, their corresponding sample sizes were significantly smaller than those in the present work, especially with thicknesses of only 0.39 mm and 1.5 mm. The reduced dimensions of the samples inherently result in a diminished probability of defects, consequently elevating the *d_33_*. In contrast, the t‐20 ceramic printed in this work by optimizing the interlayer bonding quality combines both large dimensions and excellent piezoelectric performance, with a thickness of more than 5 mm (200 layers), which is substantially greater than the preceding two samples. Concurrently, this breakthrough achievement in piezoelectric performance serves to substantiate the efficacy of the interlayer bonding quality enhancement strategy that has been proposed in the present work.

### 2.4 Piezoelectric Sensing Performance of Superlattice Component

**Figure 6:**
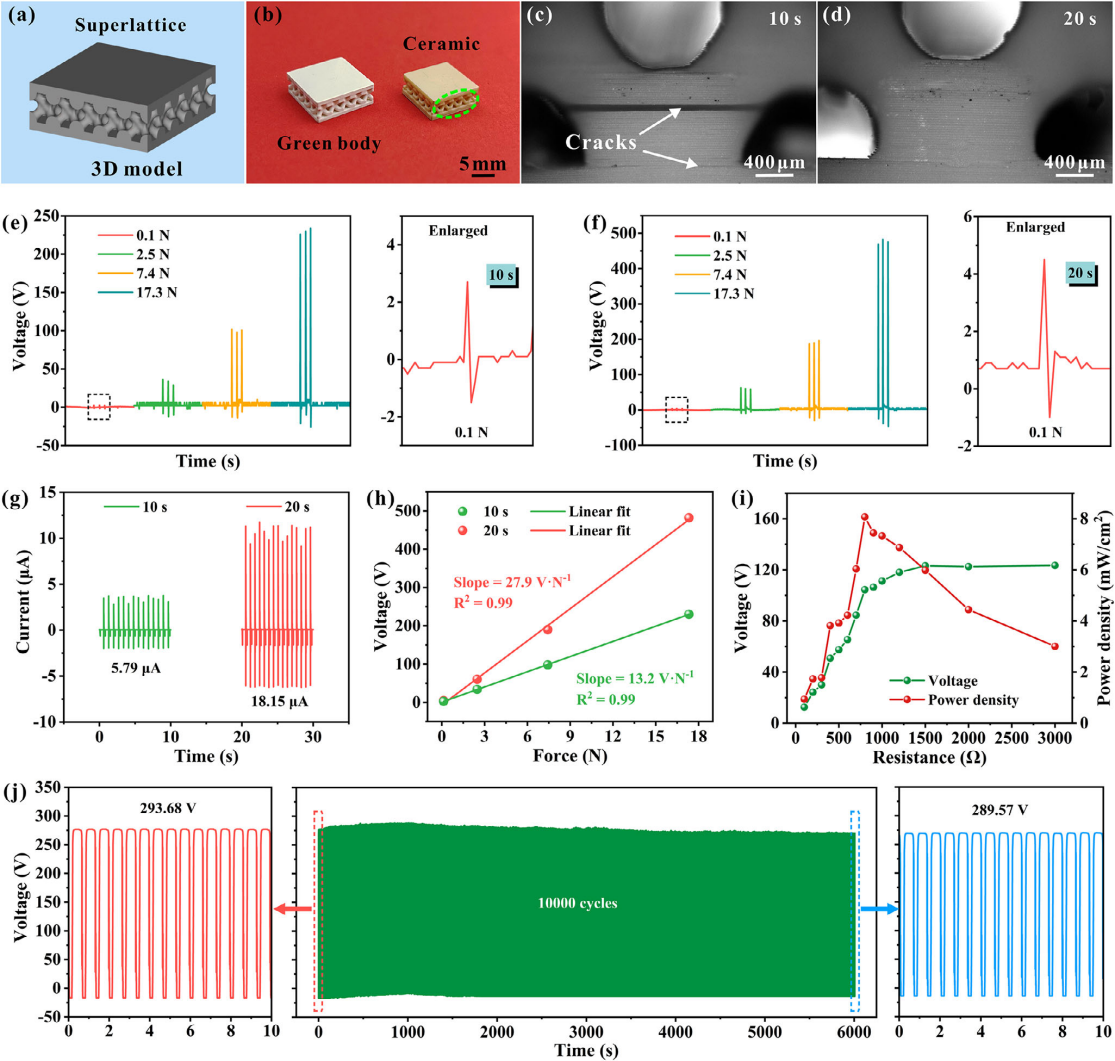
Electromechanical performance of DLP printed superlattice piezoelectric components. a,b) Superlattice piezoelectric components. c,d) Cross‐sectional optical mirror image of printed components with different exposure times. e,f) Open‐circuit voltage under different loads. g) Short‐circuit current and h) voltage sensitivity of components. i) Open‐circuit voltage and maximum power density of the components. j) Open‐circuit voltage under 10 N and 3 Hz during 10000 cycles.

**Figure** **6**a,b depicts the designed 3D model along with the printed superlattice piezoelectric component, showcasing commendable printing accuracy for both t‐10 and t‐20 components. However, detailed microstructural investigation reveals significant differences in interfacial quality between the two processing conditions. Obvious interlayer cracks are identified in the t‐10 component in Figure 6c, while the t‐20 component exhibits near flawless interlayer bonding in Figure 6d, which is consistent with the results depicted in Figure 4e. This enhanced structural integrity in the t‐20 component directly translates to superior functional performance, as demonstrated through comprehensive electromechanical characterization. The performance differential becomes particularly evident when testing the electrical output characteristics under mechanical stimulation. As illustrated in Figure 6e,f, the open‐circuit voltage of the t‐20 component exhibits a nearly twofold enhancement in response to equivalent external forces when compared to the t‐10 component. Most remarkably, the t‐20 component exhibits an open‐circuit voltage of up to 493 V and a short‐circuit current of 18.15 µA at 17.3 N in Figure 6f,g. Equally important is the component's great sensitivity to minute mechanical stimulation, it reliably generates detectable electrical signals even under very faint applied forces of 0.1 N, with a fast response time of merely 0.4 ms (Figure S17, Supporting Information). And Figure 6h depicts the voltage sensitivity of the t‐20 component attains an impressive 27.9 V·N^−1^ with a linearity of *R^2^* = 0.99, which is higher than the values of most piezoelectric sensors and energy harvesters.^[^ ^47^, ^48^, ^49^, ^50^, ^51^, ^52^ ^]^ Signifying that the piezoelectric response is capable of being maintained with high precision response across the entire pressure range. Additionally, the energy harvesting performance of the printed component was systematically evaluated through impedance matching tests. As shown in Figure 6i, under an external load of 17.3 N and a frequency of 3 Hz, the voltage steadily increases as the load resistance increases from 100 Ω to 3 kΩ. At the optimal load resistance of 800 Ω, a maximum instantaneous power density of 8.07 mW·cm^−2^ can be generated, which demonstrates remarkable energy conversion efficiency for 3D‐printed piezoelectric components. Long‐term mechanical reliability, a critical requirement for practical applications, was rigorously assessed through cyclic compression testing in Figure 6j. The t‐20 component maintains exceptional signal consistency throughout 10000 compression/release cycles at 10 N loading, with no observable degradation in open‐circuit voltage output. This outstanding stability and mechanical durability, coupled with the previously demonstrated high sensitivity and energy harvesting capability, confirms the suitability of the component for demanding piezoelectric sensing and energy harvesting applications.

**Figure 7:**
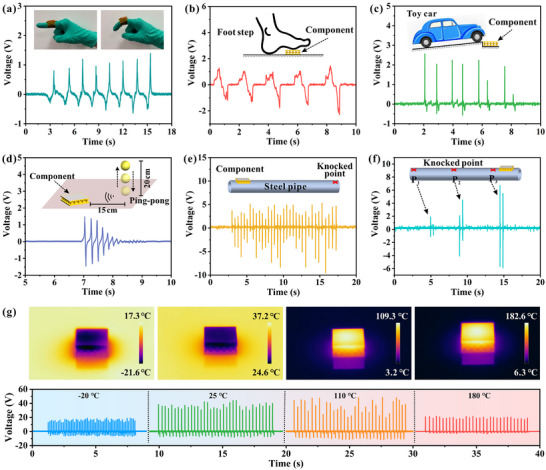
Application of printed components in different scenarios. a) Voltage response generated by knuckle bending, b) foot stepping, c) toy car pressure, d) ping‐pong ball free falling, and e) irregular knocking on the steel pipe surface. f) Voltage response caused by knocking at different positions on the steel pipe surface. g) Voltage response at different ambient temperatures.

The sensing performance of the t‐20 superlattice piezoelectric components was systematically evaluated across multiple application scenarios. As demonstrated in **Figure** **7**a–c, the components exhibit exceptional responsiveness to both subtle and substantial mechanical stimuli, generating measurable voltage signals (>1 V) across a wide dynamic range, from delicate knuckle movements to substantial loads such as toy vehicular pressure and foot impacts (Videos S1 and S2, Supporting Information). This versatility confirms their suitability for wearable electronics and pressure‐sensing applications. In disciplines such as aerospace and water engineering, there is a requirement for non‐contact dynamic monitoring of specific components, including wings and pipes, to ensure system safety, prevent equipment failures, and optimize its performance. A table tennis impact test was developed to simulate the non‐contact sensing characteristics of components in practical scenarios of structural health monitoring. The configuration of the system is demonstrated in Figure 7d, where the voltage signal of the ping‐pong ball was recorded during its descent from a height of 20 cm to the tabletop, with the drop point positioned at a distance of 15 cm from the printed component. The gradual decrease in bouncing height is accompanied by a corresponding decrease in signal intensity (Video S3, Supporting Information), suggesting that the component has robust dynamic monitoring capability. Figure 7e,f displays the non‐contact monitoring of the vibration on the steel pipe surface by the printed component. As depicted in Figure 7e, the voltage signals obtained when the steel pipe is knocked irregularly exhibit a disorganized pattern. As the knocked points approach the component (100 cm (P_1_), 50 cm (P_2_), and 5 cm (P_3_)), the voltage signals undergo a significant increase from 1.9 to 6.7 V, in Figure 7f. This discovery indicates that the component exhibits sensitivity to vibrations on the steel pipe surface, and the source localization of the object surface can be readily accomplished based on the location of the vibration points. What's more, the engineering applications of piezoelectric sensing components frequently encounter extreme working conditions, including low or high temperatures. Consequently, it is imperative to assess the piezoelectric response characteristics of the components across a broad temperature range. Figure 7g indicates that the output voltage of the printed components at −20, 25, 110, and 180 °C is 18.9, 41.2, 43.6, and 21.3 V, respectively. The decline in electrical properties of PZT ceramics at both low and elevated temperatures can be ascribed to the inherent properties of the piezoelectric materials. Although the piezoelectric response of the printed component in harsh environments decreased by nearly half compared to those at room temperature, it still exhibits a significant voltage response to external forces. This finding underscores the potential of the printed component to function over a broad range of temperatures while retaining satisfactory sensing capability.

## 3 Conclusion

This study introduces an innovative strategy to optimize interlayer bonding in DLP‐printed PZT ceramics, enabling the fabrication of piezoelectric components with superior performance and ultrahigh sensitivity. The key mechanism involves secondary curing, modulated by exposure time, which critically influences interfacial integrity. An optimal exposure duration modifies the surface properties of cured layers, improving wettability and facilitating uniform slurry recoating, thereby minimizing defect formation. Insufficient exposure time compromises curing depth and secondary curing efficacy, while excessive exposure induces excessive resin conversion and shrinkage stress, both exacerbating interlayer delamination. The t‐20 ceramic with optimal interlayer bonding quality achieves a dense microstructure, characterized by a density of 7.425 ± 0.013 g·cm^−3^. Remarkably, the ceramic piezoelectric constant (*d_33_*) reaches an impressive 516 ± 8 pC·N^−1^, rivaling commercial ceramics and surpassing most 3D‐printed advanced piezoelectric materials. The resulting superlattice component demonstrates exceptional electromechanical response, with an open‐circuit voltage of up to 493 V, short‐circuit current of 18.15 µA, and power density of 8.07 mW·cm^−2^. The component depicts an extremely pressure‐sensitive sensitivity of 27.9 V·N^−1^ and achieves a voltage response at a very faint load of 0.1 N. Furthermore, the stability of the electromechanical performance is demonstrated through 10000 compression/release cycles. Validated across diverse applications, the t‐20 component exhibits robust dynamic monitoring and sensing capabilities. These breakthroughs establish a new paradigm for additive manufacturing of high‐performance piezoelectric ceramic sensors.

## 4 Experimental Section

### Raw Materials

The commercial PZT powder, BYK‐111 dispersant, and anhydrous ethanol were combined and ball‐milled for a period of 8 h to obtain a modified powder. The dispersant constituted 1 wt% of the powder mass. Mixing of 1,6‐hexanediol diacrylate (HDDA), diphenyl tripropylene glycol diacrylate (TPGDA), and urethane acrylate (U600) resulted in the formation of a photopolymerising resin. Phenylbis (2,4,6‐trimethylbenzoyl) phosphine oxide (Irgacure 819) was employed as the photoinitiator. The modified powder was blended with the photopolymeric resin and homogenized with an electric stirrer at 1000 rpm/min to prepare PZT slurries with 60 vol.% ultra‐high solid loading.

### 3D Printing

The printing process was conducted using a DLP printer (AUTOCERA‐L40, Beijing Shiwei Technology Co., Ltd., Beijing, China) with a UV light source wavelength of 405 nm. The PZT slurries were added to the barrel of the printer and uniformly coated on the release film surface with a scraper at a spreading thickness of 50–75 µm. The printing layer thickness was set at 25 µm, and the exposure power was 7.5 mW cm^−1^ ^2^. The exposure times were 5 s, 10 s, 15 s, 20 s, 30 s, and 50 s, respectively, and the corresponding samples were designated t‐5, t‐10, t‐15, t‐20, t‐30, and t‐50. The preparation process of piezoelectric ceramics and superlattice components is shown in Figure 1a.

### Viscosity Measurement

Viscosity tests were conducted on the ceramic slurry using a rotational rheometer (MARS 40, Germany). The shear rate was set at 0–400 s^−1^, and the test temperature was maintained at 25 °C.

### Curing Depth

The Jacobs Method was used to measure the curing depth of the slurry under different exposure times (energies). The gel point of the slurry is obtained by using the UV rheological analysis, that is, the shortest exposure time for slurry curing. Prepare a single‐layer slurry film (thickness 10–50 µm) and cure it by stepwise exposure energy (30‐300 mJ·cm^−2^). A contact thickness gauge is used to measure the cured layer thickness under different exposure energies. The shrinkage of the printed ceramics was measured by means of a vernier caliper.

### Structural Characteristic Tests

A laser particle size meter was employed for the analysis of the particle size distribution of the PZT powders. An X‐ray diffractometer was used to characterize the modified PZT powder, with a test range of 2θ = 20–80°. A field emission scanning electron microscope (FE‐SEM, GeminiSEM360) and a high‐resolution transmission electron microscope (HR‐TEM, JEM‐2100F) were utilized to observe the microscopic morphology of the PZT powders and the printed ceramics. A contact angle tester (JC2000D2G, China) was used to characterize the wettability of the cured layer surface. The surface potential and roughness of the cured layer were measured using a Kelvin piezoelectric force microscope (KPFM, Dimension Icon, USA).

### Mechanical Properties Characterization

A nanoindentation tester (G200, USA) and a dynamic thermo‐mechanical analyzer (DMA Q800, USA) were employed to test the mechanical properties of green bodies. The interlayer bond strength of printed green bodies was tested using a CMT5105 series microcomputer‐controlled electronic universal testing machine.

### Chemical Bond Analysis

The unsaturated double bond conversion was characterized by Raman spectroscopy (HR Evolution, France). Chemical bond analysis of the cross‐section of printed green bodies using FTIR spectroscopy (Thermo Fisher‐Nicolet iN10).

### Thermal Analysis

A simultaneous thermal analyzer (TA‐SDT Q600, USA) was applied to examine the debinding process of the green bodies in an air atmosphere. The temperature range was set between room temperature and 600 °C, with a heating rate of 10 °C·min^−1^.

### Electrical Performance Tests

The printed piezoelectric components were subjected to a polarization process in an oil bath at 110 °C with an electric field of 35 kV·cm^−1^ for 45 min before to the electrical property tests. A quasi‐static *d_33_/d_31_* (ZJ‐6A, IACAS, China) and a ferroelectric analyzer (TF Analyzer 3000) were employed to examine the piezoelectric and ferroelectric properties of the printed ceramics, respectively. The impedance and phase angle were assessed utilizing an impedance analyzer (Agilent 4294A, Santa Clara, CA). A digital source meter (Keithley 6517, USA) was employed to quantify the output voltage and current of the piezoelectric components.

### Statistical Analysis

The results in this manuscript are presented as the mean ± standard deviation over triplicate readings (n = 3), unless specified otherwise in the figure descriptions. Origin software was used to perform peak detection, fitting, and other preprocessing of the Raman and FTIR data.

## Conflict of Interest

The authors declare no conflict of interest.

## Supporting information



Supporting Information

Supplemental Video 1

Supplemental Video 2

Supplemental Video 3

## Data Availability

The data that support the findings of this study are available from the corresponding author upon reasonable request.
