# Supplementary material for: Enhancing Interlayer Bonding in DLP‐Printed Piezoelectric Ceramics via Controlled Secondary Curing for High Piezoelectric Performance
Source: Adv Sci (Weinh). 2025 Sep 4;12(44):e12767. doi: 10.1002/advs.202512767 (PMC12667449; doi:10.1002/advs.202512767)
Supplement: Supplementary file 1 — Supporting Information [file ADVS-12-e12767-s004.docx]

**Supporting Information**

**Enhancing Interlayer Bonding in DLP-Printed Piezoelectric Ceramics via Controlled Secondary Curing for High Piezoelectric Performance**

*Yaoting Zhao, Ruihang Liu, Wenlong Wang, Jin Zhang, Hongya Liu, Wei Gao, Xiujuan Lin *, Hang Luo, Shifeng Huang *,* *and Dou Zhang **

Y. Zhao, R. Liu, J. Zhang, H. Liu, W. Gao, X. Lin, S. Huang

Shandong Provincial Key Laboratory of Green and Intelligent Building Materials, University of Jinan, Jinan, 250022, China

E-mail: [mse_linxj@ujn.edu.cn](mailto:mse_linxj@ujn.edu.cn); [mse_huangsf@ujn.edu.cn](mailto:mse_huangsf@ujn.edu.cn)

W. Wang

Naval Submarine Academy, Qingdao, 266199, China

H. Luo, D. Zhang

State Key Laboratory of Powder Metallurgy, Central South University, Changsha 410083, China

E-mail: [dzhang@csu.edu.cn](mailto:dzhang@csu.edu.cn)

The Beer-Lambert equation is as follows:

$C_{d}=D_{p}\ln\left( \frac{E_{i}}{E_{c}} \right)=D_{p}\ln E_{i}-D_{p}\ln E_{c}$ S (1)

where *C_d_* stands for the curing depth (μm), *D_p_* the penetration depth (μm), *E_i_* the surface energy density (mJ·cm^-2^), and *E_c_* indicates the critical exposure energy density (mJ·cm^-2^).


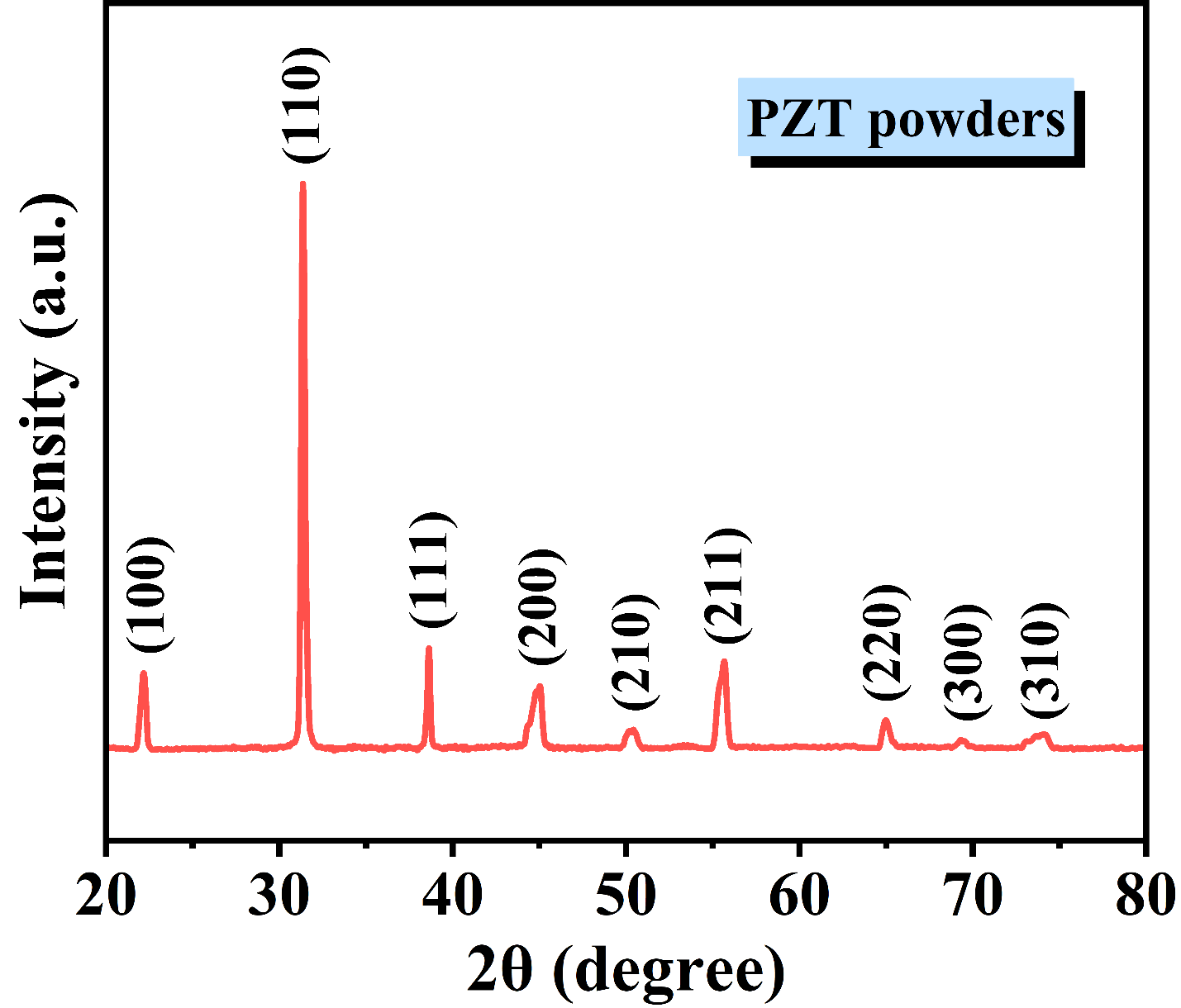


**Figure S1.** The XRD spectra of modified PZT powder.


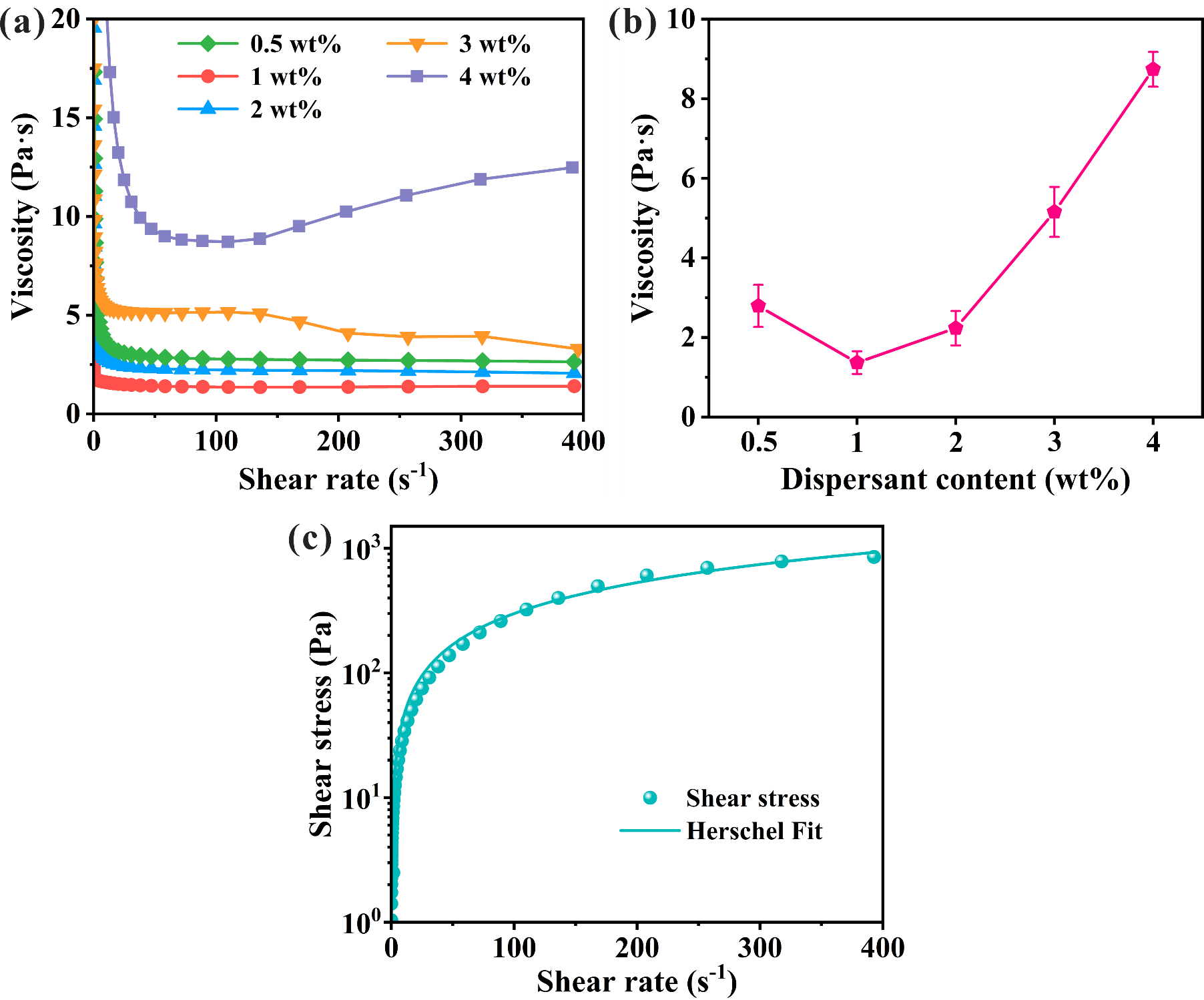


**Figure S2.** Rheological behavior of PZT slurry. (a, b) Viscosity of PZT slurries with different dispersant contents. (c) Shear stress of the slurry with 1 wt% BYK-111 dispersant.


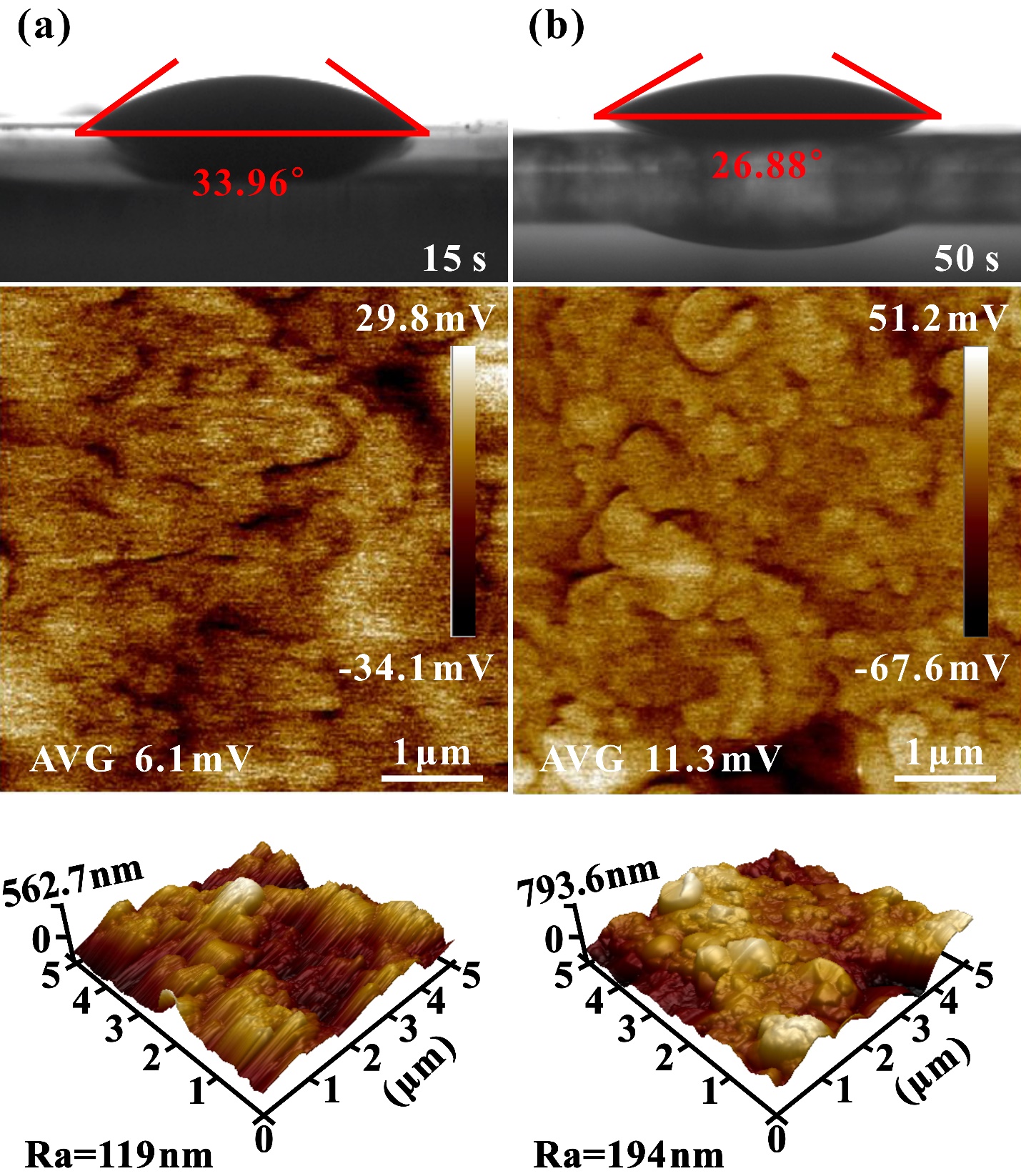


**Figure S3.** Contact angle, surface potential, and surface roughness of cured layers with different exposure times: (a) 15 s, (b) 50 s.


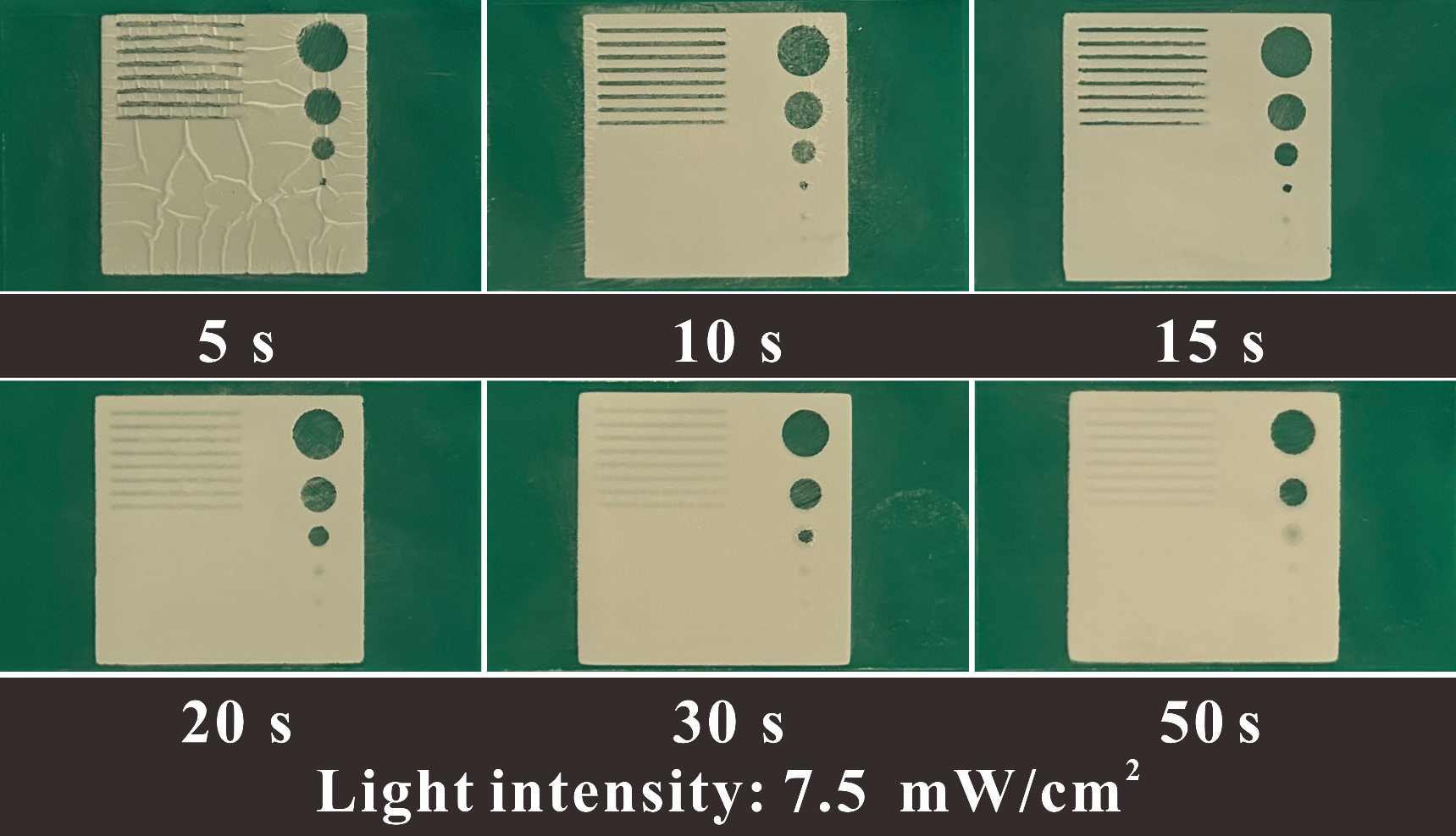


**Figure S4.** Printing accuracy of the slurry at different exposure times.


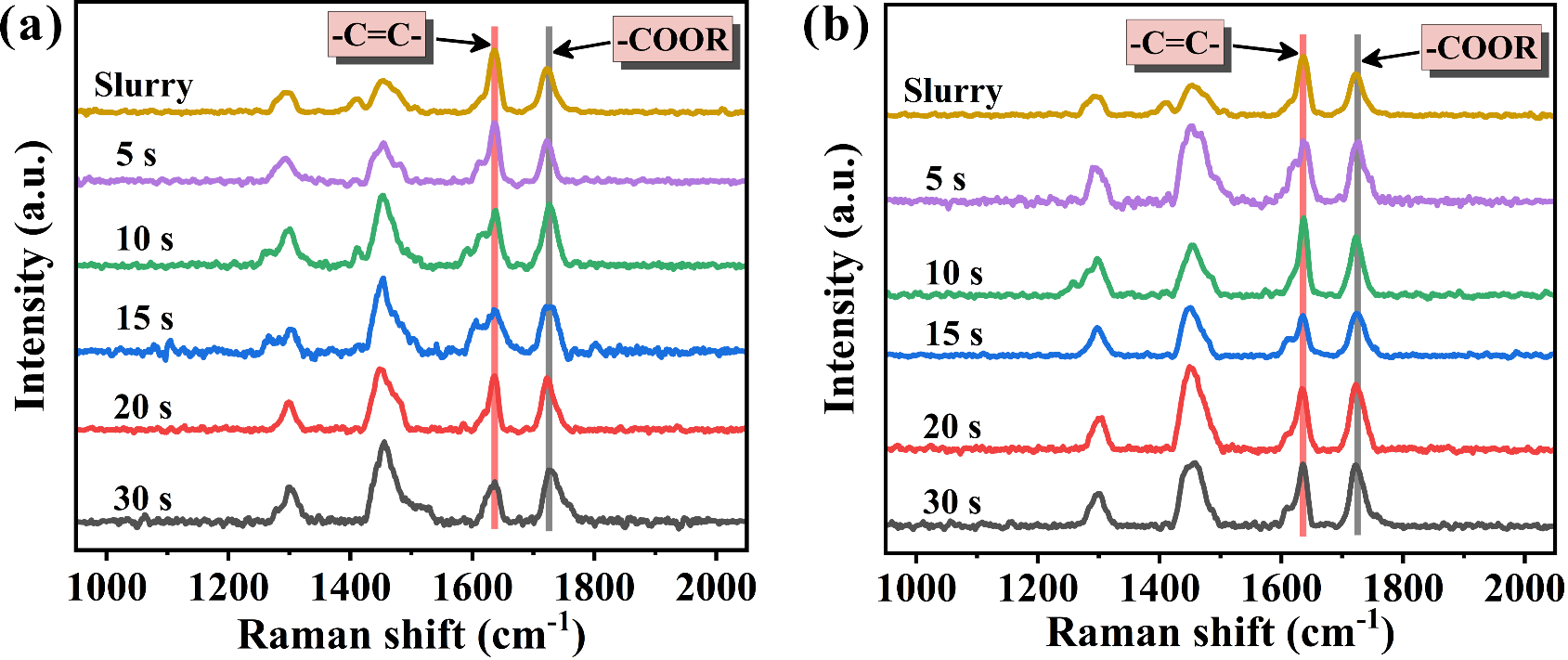


**Figure S5.** Raman spectra of the green bodies cross-sections at (a) Surface (R1) and (b) intra-layer (R2).


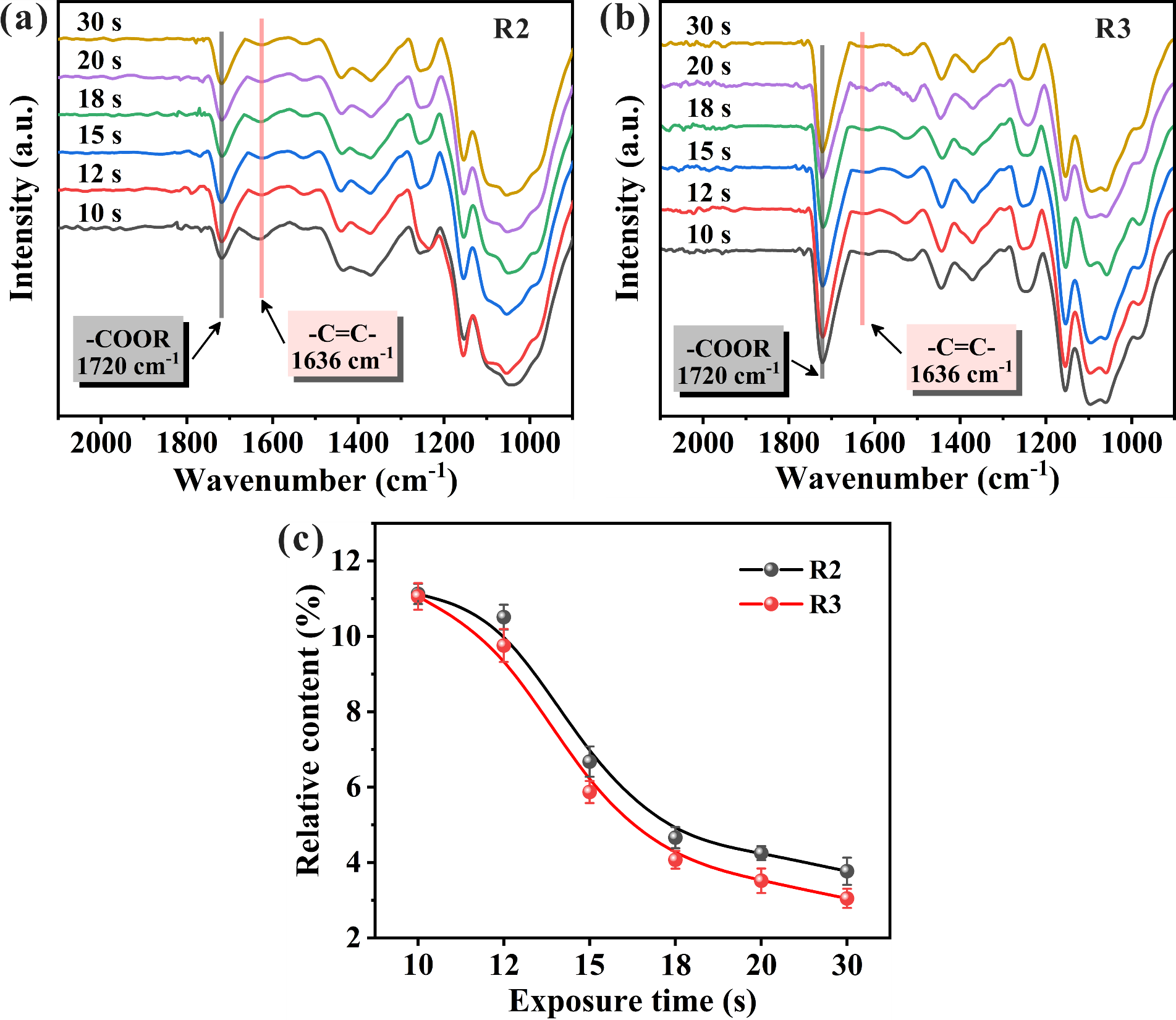


**Figure S6.** FT-IR results for printed green bodies with different exposure time (a) and relative content of double bond (b).


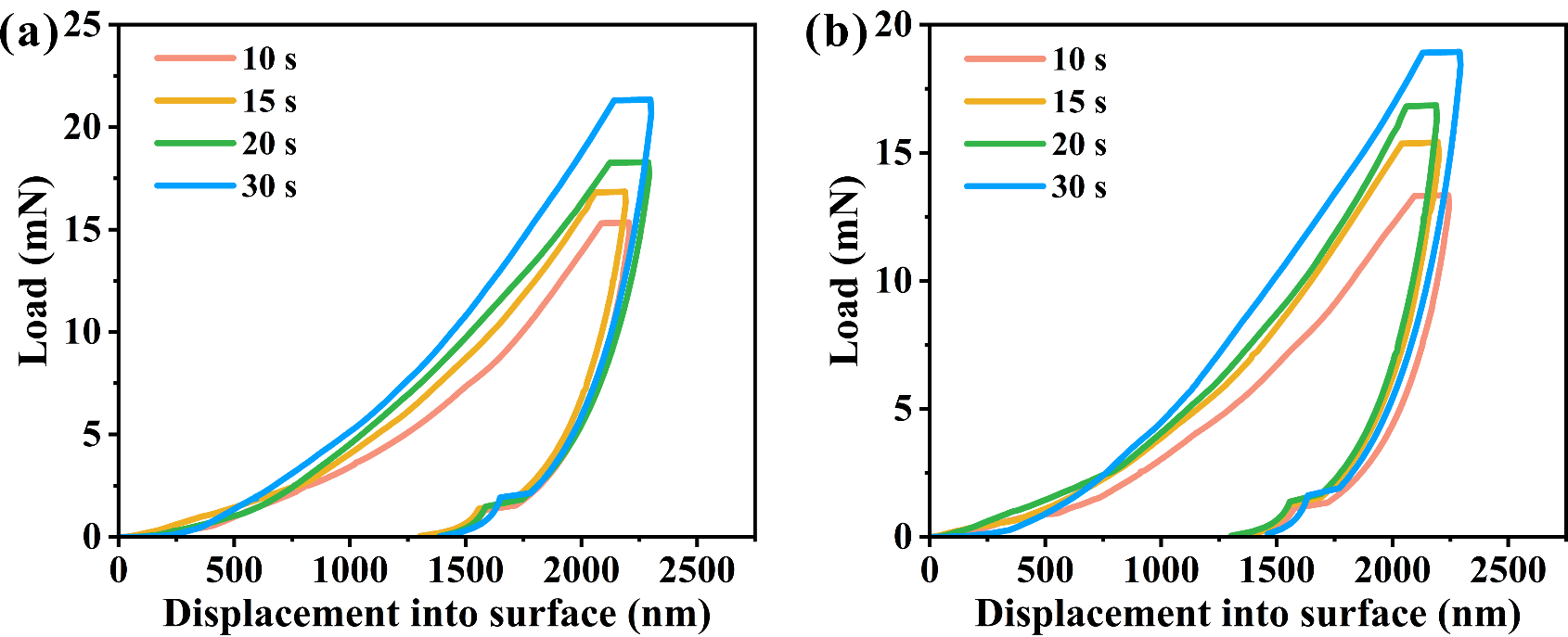


**Figure S7.** Displacement-load curves at (a) Surface (R1) and (b) intra-layer (R2) on the cross-section of green bodies.


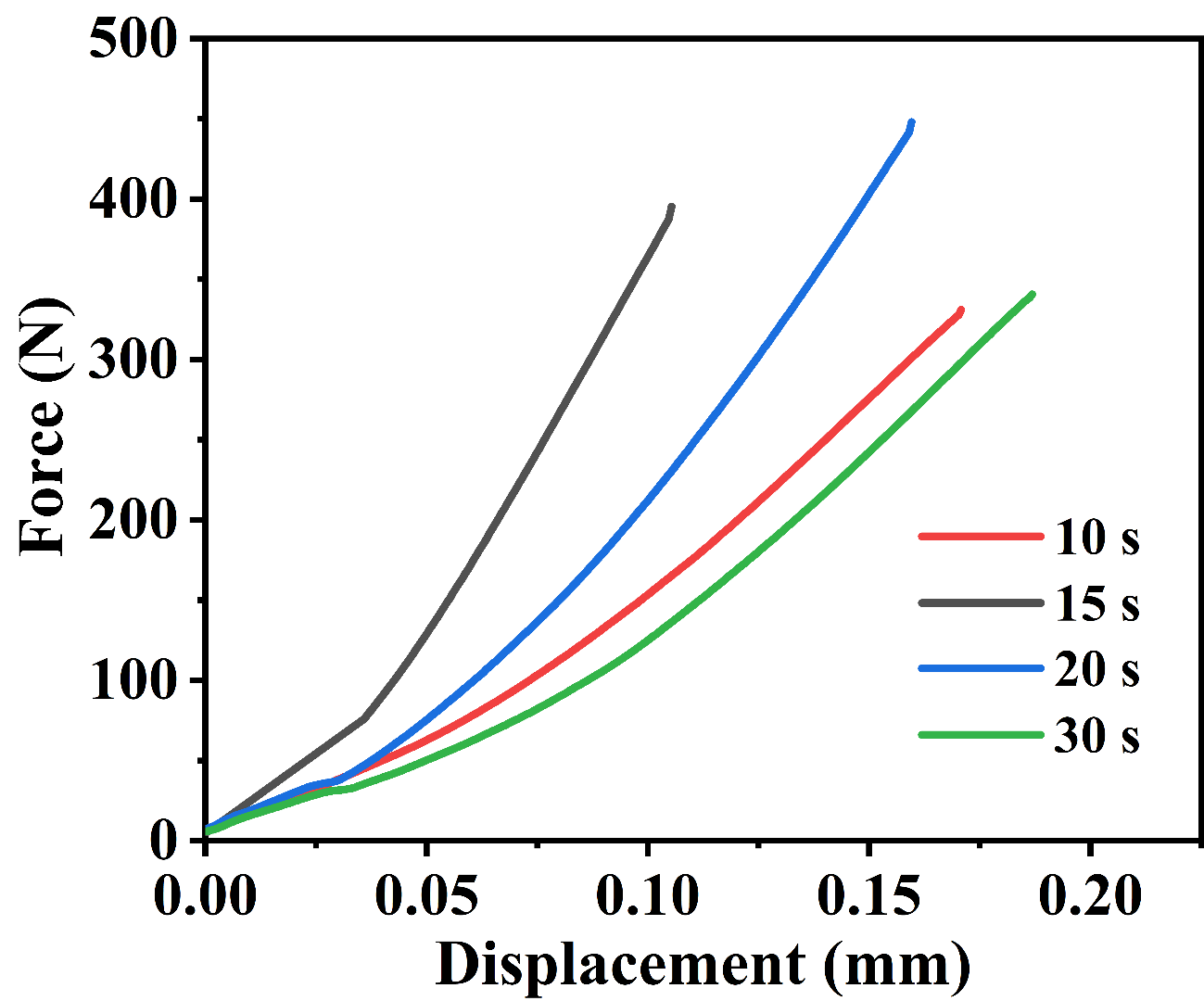


**Figure S8.** Displacement-Force curves of printed green bodies.


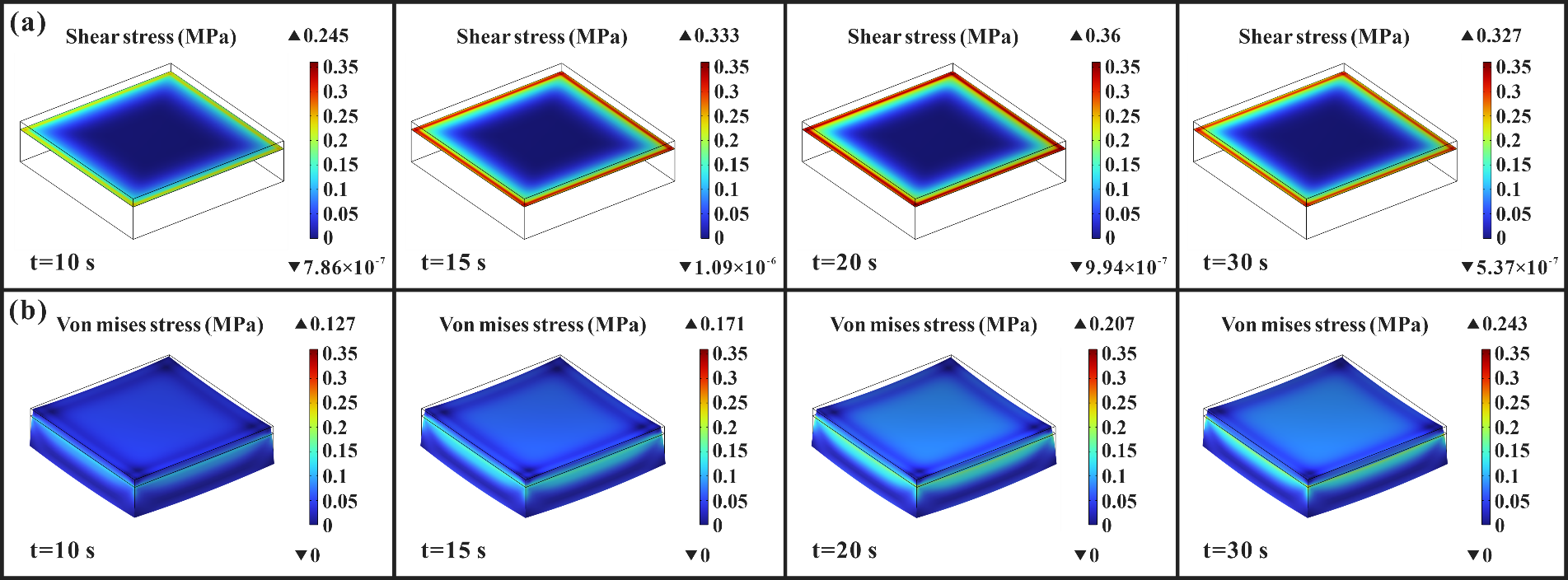


**Figure S9.** Finite element simulation results of interlayer shear strength (a) and residual stress (b) of printed green bodies.


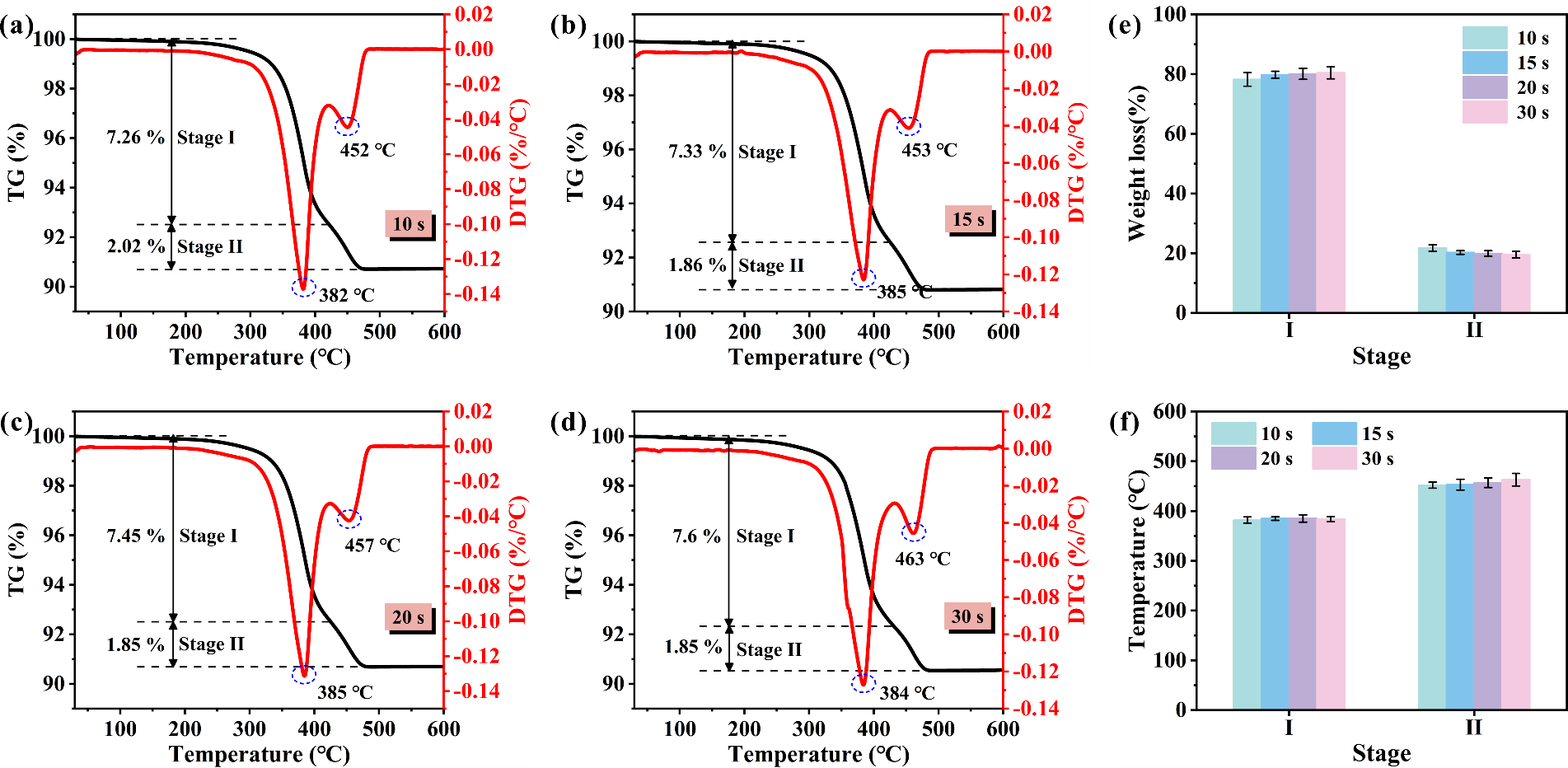


**Figure S10.** Thermogravimetric analysis results of printed green bodies. (a-d) TG-DTG curves of green bodies with different exposure times. (e) Stage I and Stage II of mass loss. (f) Temperatures of peak mass loss for both stages.

~~
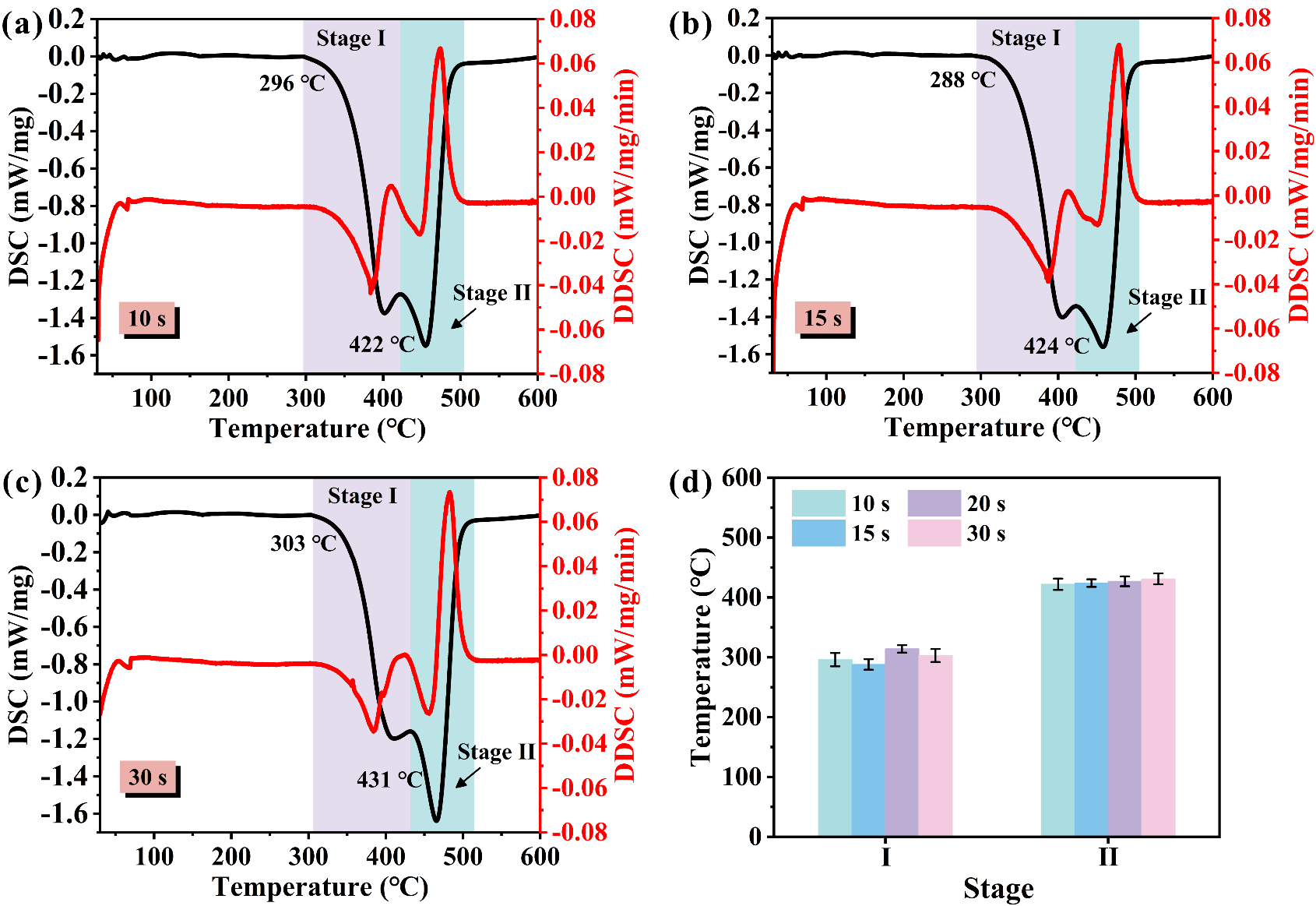
~~

**Figure S11.** Differential scanning calorimetry (DSC) results of printed ceramics. (a-c) DSC-DDSC curves of ceramics with different exposure times. (d) Peak temperatures of the thermal effects for Stage I and II.

The temperature was increased from room temperature to 600 °C at a ramp rate of 0.2 °C·min^-1^, where the temperature was held for 2 h each at 385 ℃ and 465 °C and for 1 h at 600 °C to ensure complete decomposition of the organics. It was imperative to reduce the rate of heating during the degreasing phase in order to ensure that the organics in the green bodies were heated uniformly. Meanwhile, this approach served to eliminate interlayer defects caused by thermal stress. The PZT ceramics were sintered by gradually increasing the temperature from 600 ℃ to 1180 °C at a rate of 5 °C·min^-1^ and holding it for 2 h.


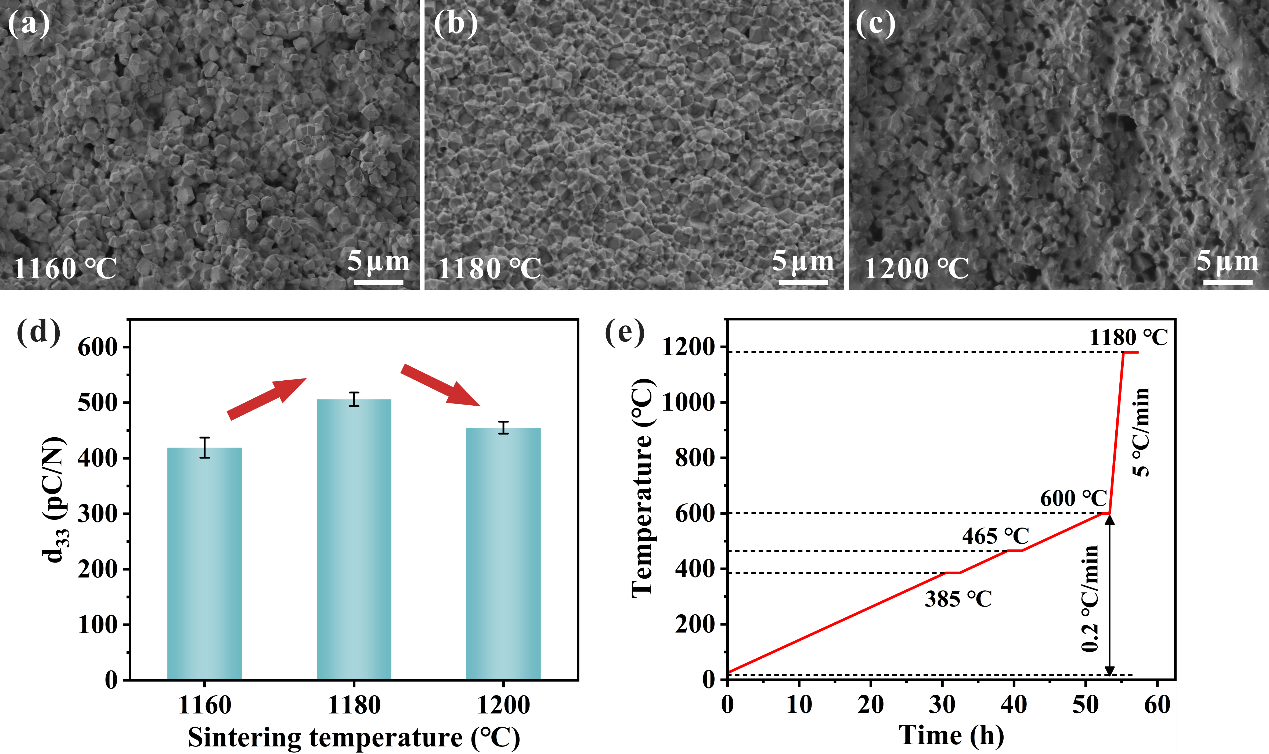


**Figure S12.** Sintering temperature adjusting of printed PZT ceramics. (a-c) Microstructure and (d) piezoelectric constant d_33_ of printed ceramics at different peak sintering temperature. (e)The sintering process of the printed PZT ceramics.


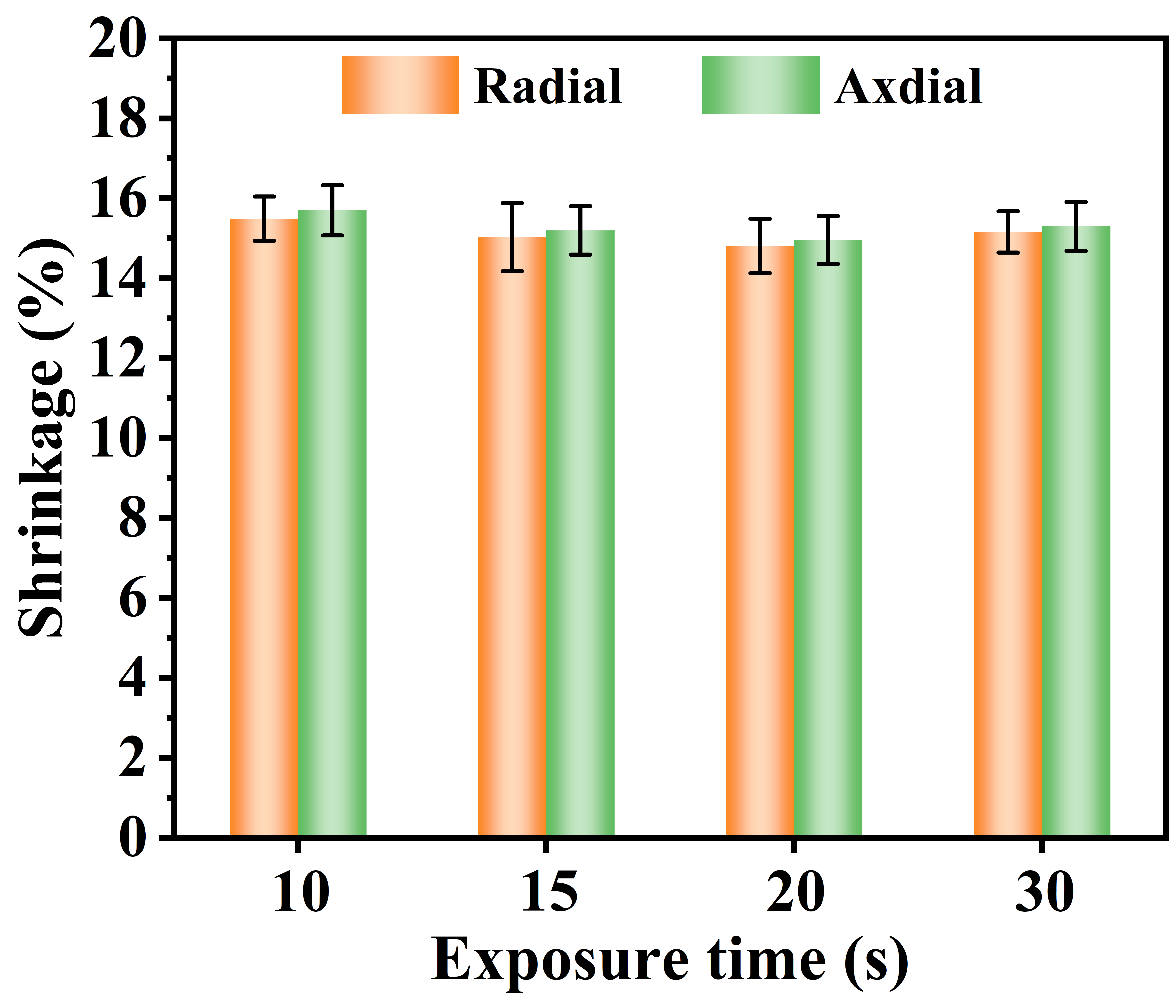


**Figure S13.** The shrinkage of printed ceramics.


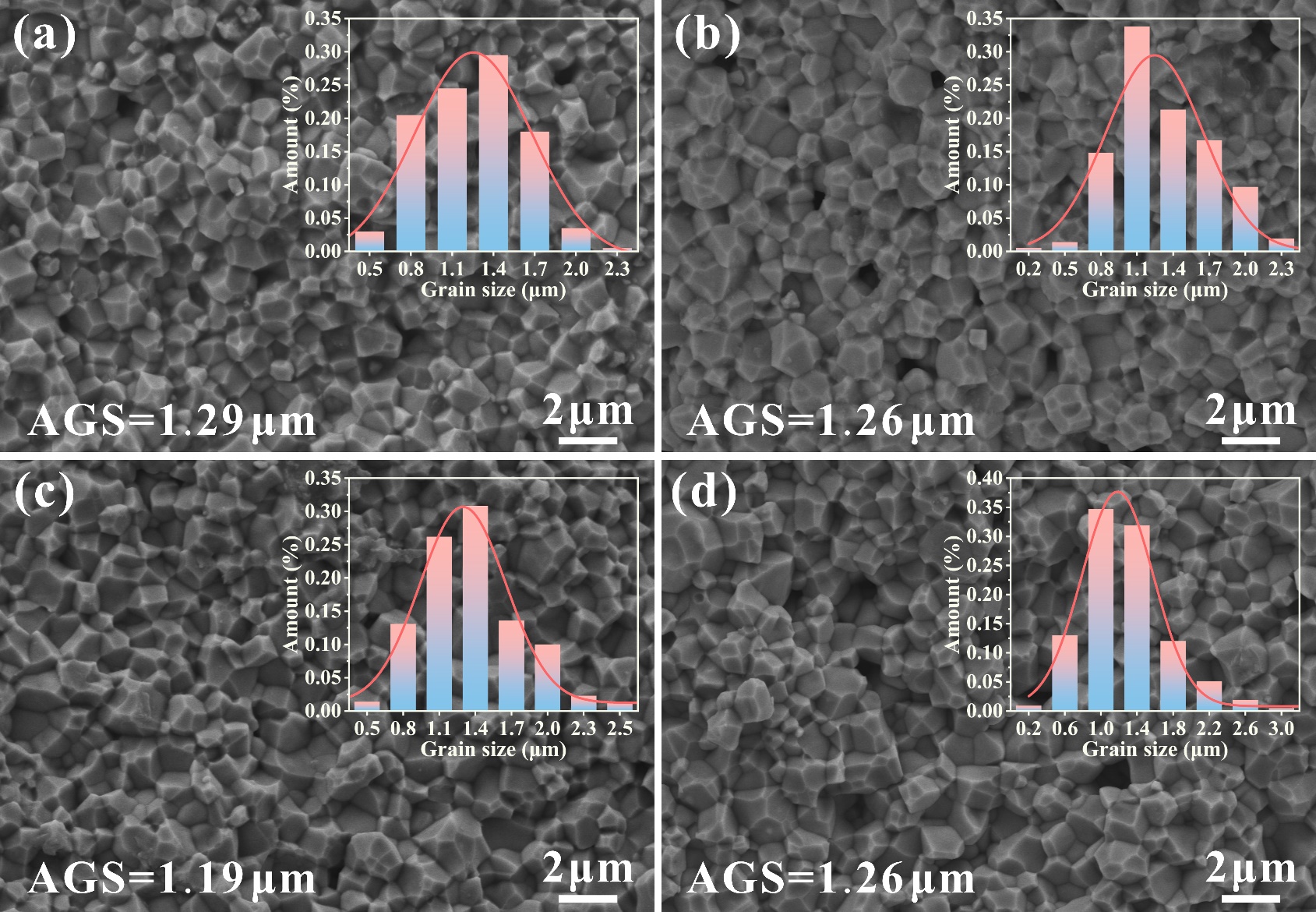


**Figure S14.** Microstructure of printed ceramics with different exposure times: (a) 10 s, (b) 15 s, (c) 20 s, (d) 30s.


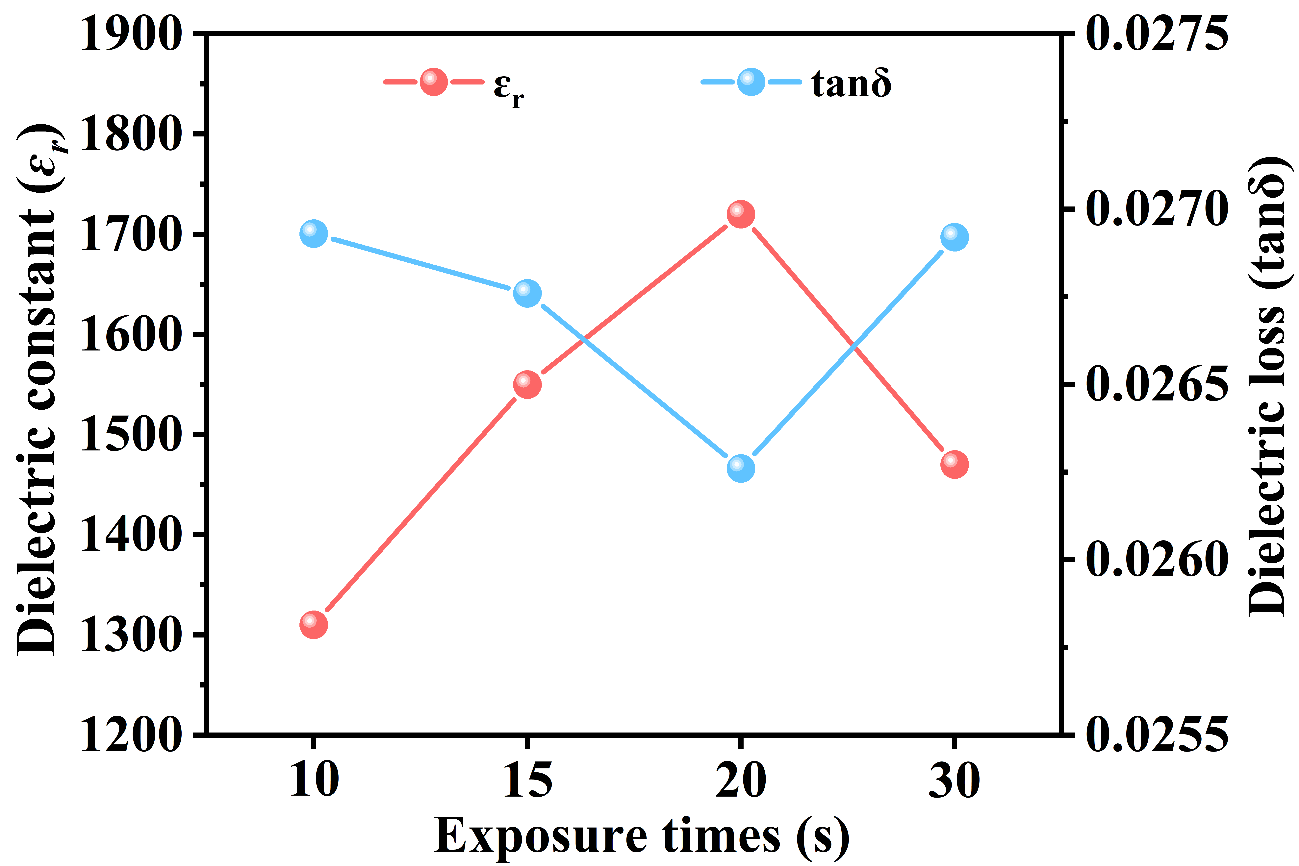


**Figure S15.** Dielectric constant (*ɛ_r_*) and dielectric loss (*tanδ*) of printed ceramics at 1 kHz.


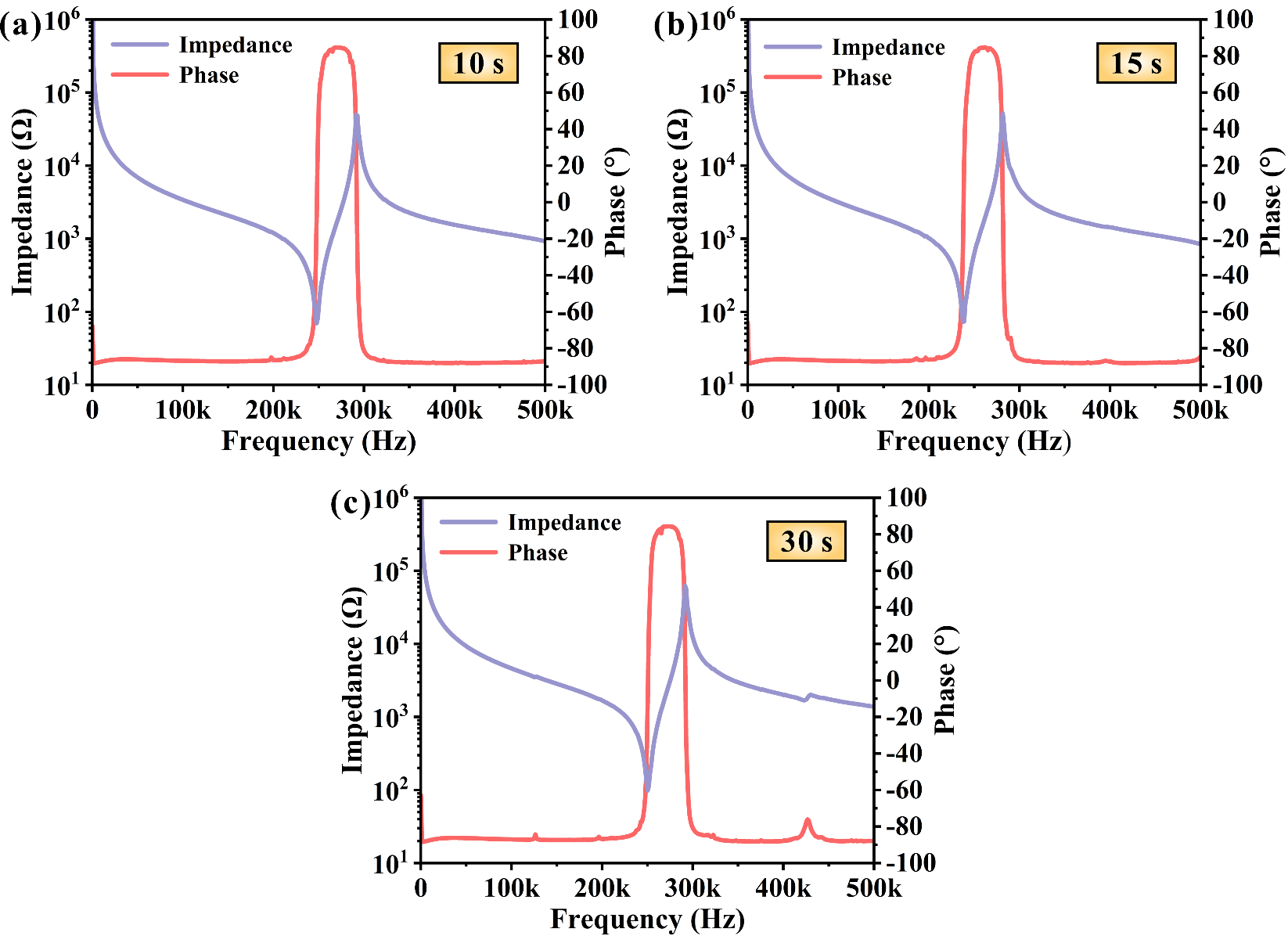


**Figure S16.** Impedance and phase spectrum of printed ceramics with different exposure times.


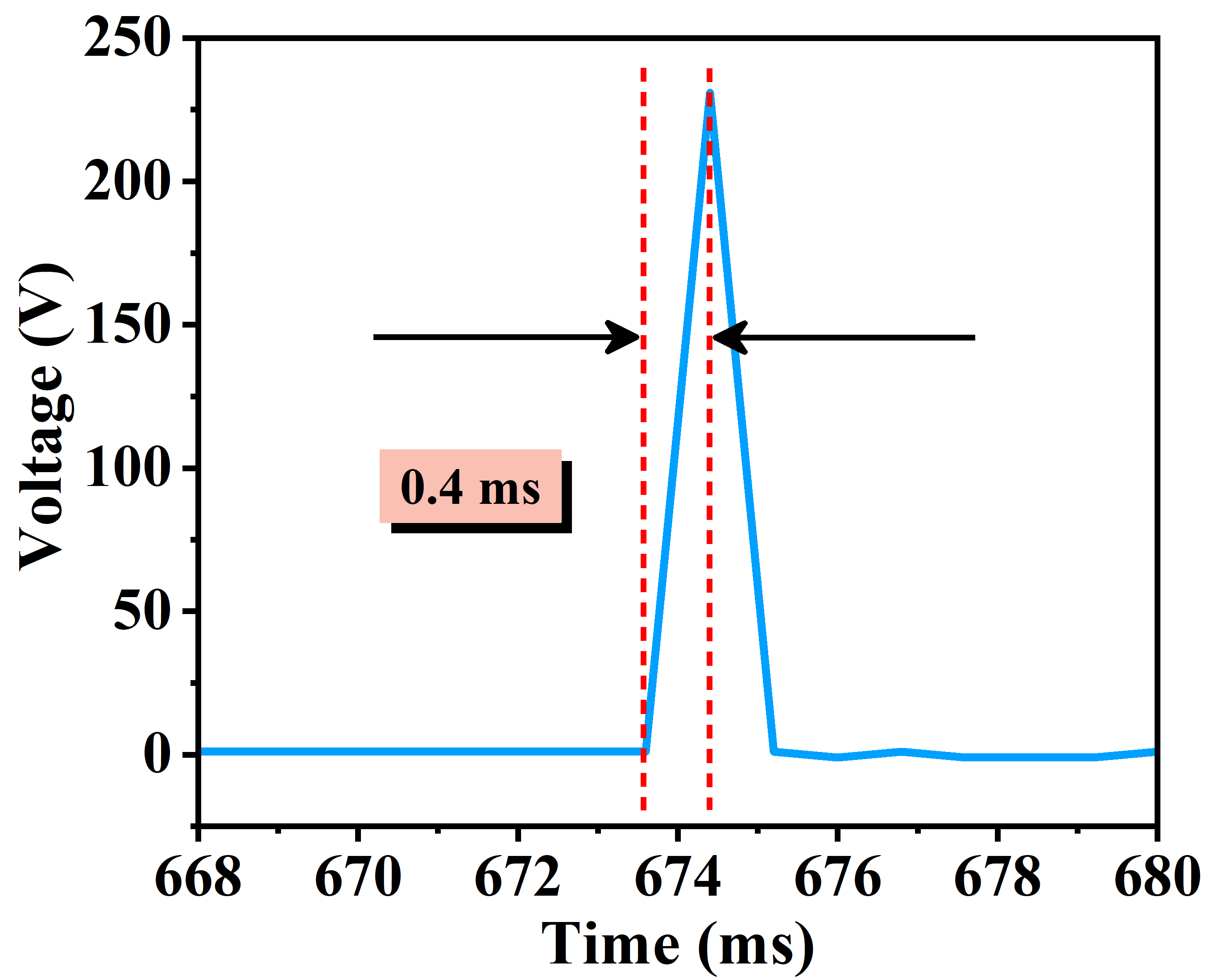


**Figure S17.** Response time of the printed superlattice piezoelectric component.
